# Supplementary material for: N-Acryloylindole-alkyne (NAIA) enables imaging and profiling new ligandable cysteines and oxidized thiols by chemoproteomics
Source: Nat Commun. 2023 Jun 15;14:3564. doi: 10.1038/s41467-023-39268-w (PMC10272157; doi:10.1038/s41467-023-39268-w)
Supplement: Supplementary file 2 — Supplementary Information [file 41467_2023_39268_MOESM2_ESM.pdf]

Supplementary Information for

N-Acryloylindole-alkyne (NAIA) enables imaging and profiling new ligandable cysteines and oxidized thiols by chemoproteomics

Tin-Yan KOO,[1,Δ] Hinyuk LAI, [1,Δ] Daniel K. NOMURA[2,3] and Clive Yik-Sham CHUNG[1,4,5,*]

[1] *School of Biomedical Sciences, Li Ka Shing Faculty of Medicine, The University of Hong Kong, Pokfulam Road, Hong Kong, P. R. China*

[2] *Department of Chemistry, University of California, Berkeley, Berkeley, CA, USA*

[3] *Department of Molecular and Cell Biology, University of California, Berkeley, Berkeley, CA, USA*

[4] *Department of Pathology, School of Clinical Medicine, Li Ka Shing Faculty of Medicine, The University of Hong Kong, Pokfulam Road, Hong Kong, P. R. China*

[5] *Centre for Oncology and Immunology, Hong Kong Science Park, Hong Kong SAR, China*

[Δ] *These authors contributed equally*

*To whom correspondence should be addressed. Email: cyschung@hku.hk

Supplementary Methods

Materials and Reagents for Chemical Synthesis

4-Hydroxyindole, 5-hydroxyindole, triphenylphosphine and sodium cyanoborohydride were purchased from AK Scientific. Acryloyl chloride, sodium hydride (60% dispersion in mineral oil), acetic acid, 4,5-dichloro-3,6-dioxo-1,4-cyclohexadiene-1,2-dicarbonitrile (DDQ), *tert*-butyl bromoacetate, potassium carbonate and *N*,*N*-diisopropylethylamine (DIPEA) were purchased from Sigma-Aldrich. Hex-5-yn-1-amine was purchased from Combi-Blocks. 85% Phosphoric acid was purchased from Alfa Aesar. CL1–CL21 and CL-Sc were purchased from Enamine LLC with purity of >95%. 2-(Methylsulfonyl)benzo[d]thiazole (MBST) and *N*-hydroxybenzimidoyl chloride, with purity of 96% and 95% respectively, were purchased from BLD Pharm. All other reagents were of analytical grade and were used without further purification. MilliQ water was used in all experiments unless otherwise stated.

Materials and Reagents for biological experiments

Azide-fluor 545, iodoacetamide, copper(II) sulfate pentahydrate, Tris(2-carboxyethyl)phosphine hydrochloride (TCEP) and DPBS were purchased from Sigma-Aldrich. *N*-Hex-5-ynyl-2-iodoacetamide was from Chess Fine Organics. Tris[(1-benzyl-1H-1,2,3-triazol-4-yl)methyl]amine (TBTA) was from Cayman Chemical. WST-8 was from Abcam (228554). FBS (35-015-CV) and 0.25% Trypsin-EDTA (1×; 25200-056) were from Gibco. DMEM (10-013-CV), GluatMax (100×; 25030-081), HyClone™ Leibovitz L-15 Media (SH30525.01) B-PER™ Bacterial Protein Extraction Reagent (78243), Pierce Protease and Phosphatase Inhibitor Mini Tablets,EDTA-free (A32961), Invitrogen™Propidium Iodide (P3566), Pierce™ Silver Stain Kit (24612), Pierce™ Glutathione Agarose (16100) and Pierce™ Streptavidin Agarose beads (20349) were from Thermo Fisher Scientific. Sequencing grade modified trypsin (V51111) and GSH/GSSG-Glo™ Assay kit (V6611) were from Promega. *N*-Acetyl-L-cysteine methyl ester (9424AL) and *N*-acetyl-L-aspartic acid (U003) were from AK Scientific. Ac-Glu-OMe (QB-0580) and Ac-Tyr-OMe•H_2O (SS-4784) were from Combi-Blocks. *N*-Acetyl-L-histidine was from Enamine LLC. *N*-Fmoc-L-proline (47636) was from Sigma Aldrich. Rac1 Pull-Down Activation Assay Biochem Kit (BK035) was obtained from Cytoskeleton. Reagents for western blot (20x LumiGLO® Reagent and 20X Peroxide (7003), GAPDH (14C10) antibody (2118), phosphor-PAK1(Thr423)/PAK2(Thr402) antibody (2601), Pak1/2/3 antibody (2604), Rac1/2/3 antibody (2465), E2f1 antibody (3742S), phospho-Rb(Ser780) Antibody (9307), cyclin D1 (92G2) antibody (2978), Rb (4H1) antibody (9309), anti-rabbit and anti-mouse IgG HRP linked antibody (7074 and 7076) were purchased from

Cell Signal Technology. TGX™ FastCast™ Acrylamide Kit (1610173) and nitrocellulose membrane (1620112) for SDS-PAGE and western blot were from Bio-Rad.

Physical Measurements and Instrumentation

^{1}H NMR and $^{13}C\{^{1}H\}$ spectra were collected at 25 °C on Bruker AVB-400, AVQ-400 and AV-300 at the College of Chemistry NMR Facility at the University of California, Berkeley, or on Bruker AVANCE NEO 600 MHz spectrometer at the Department of Chemistry at the University of Hong Kong. All chemical shifts are reported in the standard δ notation of parts per million relative to residual solvent peak as an internal reference. Splitting patterns are indicated as follows: br, broad; s, singlet; d, doublet; t, triplet; m, multiplet; dd, doublet of doublets; tt, triplet of triplets. High-resolution mass spectra were collected at the QB3/Chemistry Mass Spectrometry Facility at the University of California, Berkeley. UV-Vis absorption and fluorescence from microplates were recorded on SpectraMax i3 (Molecular Devices) or on Perkin Elmer Victor 3 (Molecular Devices). In-gel fluorescence images were recorded on ChemiDoc MP Gel Imaging system (Bio-Rad). Reaction kinetics of cysteine-reactive compounds with amino acids in aqueous buffer solution were measured by liquid chromatography-tandem mass spectrometry on a Waters Autopurification System using a SunFire C18 HPLC column (50 × 4.6 mm with 5 µm diameter particles, Waters), or an Agilent 6430 QQQ using a Luna reverse-phase C5 column (50 × 4.6 mm with 5 mm diameter particles, Phenomenex). Confocal microscopic images were recorded on a Zeiss laser scanning microscope 880 with a 20× air objective lens using ZEN 2.3 (Black Version) software (Carl Zeiss) with 3× magnification zoom-in.

***In vitro* selectivity of NAI for reacting with Cys over other amino acids.** Stock solution of NAIA-4 and NAIA-5, respectively, in DMSO was diluted by PBS/MeOH solution mixture (4:1, v/v; 500 µL), reaching final concentration of 10 µM. Stock solution of amino acid in DMSO was then freshly prepared, and added to the compound solution at final concentration of 30 µM. The solution mixture was incubated at room temperature for 30 min. After that, an aliquot of the reaction mixture (10 µL) was sent for LC-MS analysis. Selected ion chromatograms, with m/z = 325 corresponding to $[NAIA\text{-}4+H]^+$ or $[NAIA\text{-}5+H]^+$, were extracted and analyzed by integrating the area under curve. Significant decreases in NAIA-4 and NAIA-5 levels were only found in the solution mixture with *N*-acetyl-ʟ-cysteine methyl ester, indicating the high selectivity of NAIs toward reactions with Cys.

WST-8 cell viability assay of cells treated with NAIA-4 or IAA. 231MFP cells were plated on 96-well plates (Corning, 3904) at 30,000 cells per well and allowed to grow in complete medium overnight. The cells were then incubated with DMSO solvent control, NAIA-4 or IAA at indicated concentrations for 2 h. The solution was replaced with new culture medium containing 10 µL of WST-8 solution (Abcam; ab228554), and incubated for 2 h in dark at 37 °C. Cell viability were then assayed by the absorption at 460 nm on SpectraMax i3 (Molecular Devices).

MTT cell viability assay of cells treated with NAIA-5. HepG2 cells were plated on 96-well plates (Corning, 3904) at 20,000 cells per well in 100 µL of complete medium and allowed to grow overnight. The cells were then incubated with DMSO solvent control or NAIA-5 at indicated concentrations for 1 h. The treated cells were then added with 10 µL of MTT solution in PBS (5 mg/mL) and incubated in dark at 37 °C with 5% CO_2 for 4 h. After that, 100 µL of SDS solution in PBS (0.5 g/mL with 0.01M HCl) was added for cell lysis. The plates were kept in dark overnight and cell viability were assayed by the absorption at 580 nm on Perkin Elmer Victor 3 (Molecular Devices).

Stability of NAIA-5 in aqueous buffer solution over time. NAIA-5 (10 µM) was dissolved in PBS/MeOH solution mixture (4:1, v/v; 500 µL) and stay at room temperature. At predetermined time intervals, an aliquot of the reaction mixture (10 µL) was sent for LC-MS analysis on Waters Autopurification System using a SunFire C18 HPLC column (50 × 4.6 mm with 5 µm diameter particles, Waters). Separation was achieved by gradient elution from 5% to 100% MeCN in water (constant 0.1 vol % formic acid) over 4 min, isocratic elution with 100% MeCN (with 0.1 vol% formic acid) from 4 to 8 min, and returning to 5% MeCN in water (with 0.1 vol% formic acid) and equilibrated for 2 min. Selected ion chromatograms, with m/z = 325 corresponding to $[NAIA\text{-}5+H]^+$, were extracted and analyzed by integrating the area under curve. No significant changes in NAIA-5 level in the aqueous buffer solution over time was found, suggesting the high stability of NAIA-5 in the aqueous buffer solution.

MS-based ABPP experiments using Multidimensional Protein Identification Technology (MudPIT). 231MFP cells were lysed in PBS by sonication. After BCA assay and protein normalization, 231MFP cell lysates in PBS (2 mg/mL, 2 mL) were incubated with NAIA-4 and IAA (100 µM; the working concentration of IAA in many reported MS-based ABPP experiments), respectively, at room temperature for 1 h with vortexing. Then, the sample

preparation until the step of peptide elution from streptavidin beads was the same as the one described for MS-based ABPP experiment on HepG2 cell lysates.

After elution of probe-modified peptides, a fused silica capillary tubing (250 μm inner diameter) packed with 4 cm of Aqua C18 reverse-phase resin (Phenomenex no. 04A-4299) was equilibrated by a high-performance liquid chromatograph using buffer A (95:5 water:acetonitrile, 0.1% formic acid) and B (80:20 acetonitrile:water, 0.1% formic acid) with the gradient from 100% buffer A to 100% buffer B over 10 min, followed by a 5 min wash with 100% buffer B and a 5 min wash with 100% buffer A. Then, the eluted probe-modified peptides were pressure-loaded onto the capillary tubing. The tubing containing the peptide samples were then attached using a MicroTee PEEK 360 μm fitting (Thermo Fisher Scientific no. p-888) to a 13 cm laser pulled column packed with 10 cm Aqua C18 reverse-phase resin and 3 cm of strong-cation exchange resin. Samples were analyzed using an Q Exactive Plus mass spectrometer (Thermo Fisher Scientific) with a five-step Multidimensional Protein Identification Technology (MudPIT) program, using 0, 25, 50, 80 and 100% salt bumps of 500 mM aqueous ammonium acetate and using a gradient of 5–55% buffer B in buffer A. Data were collected in data-dependent acquisition mode with dynamic exclusion enabled (60 s). One full mass spectrometry (MS1) scan (400–1,800 mass-to-charge ratio (m/z)) was followed by 15 MS2 scans of the nth most abundant ions. Heated capillary temperature was set to 200 °C and the nanospray voltage was set to 2.75 kV.

Data was searched against the Uniprot human database using ProLuCID search methodology in IP2 v.3 (Integrated Proteomics Applications, Inc.).[S1] Cysteine residues were searched with a static modification for carbamidomethylation (+57.02146) and up to two differential modifications for methionine oxidation, and cysteine modification by NAIA-4 or IAA (+681.38500 or +494.32167). Only fully tryptic digested peptides were analyzed. ProLuCID data was filtered through DTASelect to achieve a peptide false-positive rate below 5%.

Competitive gel-based ABPP experiments to discover covalent ligands targeting ligandable cysteines probed by NAIA-5 or IAA. 50 μL of HepG2 cell lysates (2 mg/mL) in PBS were incubated with DMSO control or covalent ligands (50 μM) for 1 h at room temperature. Then, the solution mixtures were incubated with NAIA-5 (0.1 μM) or IAA (1 μM) for 1 h at room temperature. A master mix for CuAAC were prepared from azide-fluor 545 (5 mM), copper(II) sulfate (9.5 mM), TBTA (1 mM) and freshly prepared TCEP (50 mM), and

added to the lysates with the final concentrations of azide-fluor 545, copper(II) sulfate, TBTA and TCEP in the solution mixture at 25 µM, 1 mM, 100 µM and 1 mM respectively. The solution was incubated in dark at room temperature with shaking for 1 h, and then the reaction was quenched with 4× reducing Laemmli SDS sample loading buffer (Alfa Aesar) and heated at 90 °C for 5 min. Samples were then separated by molecular weight on precast 4–20% Tris-Glycine Plus gels (Thermo Scientific) and scanned by ChemiDoc MP (Bio-Rad Laboratories, Inc) for measuring in-gel fluorescence.

Competitive gel based ABPP with Rac1 protein. GST-Rac1 (30 µg) was treated with DMSO or CL1 at indicated concentration for 1 h at 37 °C, followed by incubation with NAIA-5 (0.1 µM) for another hour at room temperature. Gel-based ABPP experiment was performed as described above. After imaging in-gel fluorescence, the gel was stained with Pierce™ Silver Stain Kit (24612) to measure protein loading.

Synthesis

1a. K_2CO_3 (9.34 g, 67.6 mmol) was added to 4-hydroxyindole (3 g, 22.5 mmol) and *tert*-butyl bromoacetate (5 mL, 33.8 mmol) in acetone (100 mL). The solution was heated under reflux overnight. After the reaction, undissolved solid was filtered off, and the filtrate was evaporated under reduced pressure. The crude product was purified by column chromatography on silica gel using hexane/ethyl acetate (8:1, v/v) as eluent, yielding **1a** as an off-white solid (3.27 g, 59%). ^{1}H NMR ($CDCl_3$, 400 MHz): δ = 8.54 (1H, s), 7.00-7.13 (2H, m), 6.99 (1H, t, J = 2.7 Hz), 6.72 (1H, t, J = 2.2 Hz), 6.48 (1H, d, J = 7.6 Hz), 4.74 (2H, s), 1.56 (9H, s). ^{13}C{^{1}H} NMR ($CDCl_3$, 100 MHz) δ = 168.7, 151.5, 137.5, 123.3, 122.1, 118.7, 105.6, 100.7, 99.5, 82.3, 66.1, 28.0. MS (ESI$^+$): *m/z* 248 ([M+H]$^+$).

1b. The procedure was similar to that of **1a** except 5-hydroxyindole (3 g, 22.5 mmol) was used instead of 4-hydroxyindole. The crude product was purified by column chromatography on silica gel using hexane/ethyl acetate (8:1, v/v) as eluent, yielding **1b** as an off-white solid (3.8 g, 68%). ^{1}H NMR ($CDCl_3$, 500 MHz): δ = 8.12 (1H, s), 7.28-7.30 (1H, m), 7.19 (1H, t, J = 2.8 Hz), 7.07 (1H, d, J = 2.30 Hz), 6.93 (1H, dd, J = 2.45 and 8.78 Hz), 6.47 (1H, s), 4.55 (2H, s), 1.50 (9H, s). MS (ESI$^+$): *m/z* 248 ([M+H]$^+$).

2a. 1a (2.58 g, 10.4 mmol) was dissolved in acetic acid (30 mL). $NaBH_3CN$ (1.96 g, 31.3 mmol) was added to the solution mixture portionwise at 10 ^oC. The solution mixture was then stirred at 10 ^oC for 4 h, and then water was added to quench the reaction. Any organic volatile was removed by evaporation under reduced pressure, and the aqueous layer was extracted with dichloromethane. The dichloromethane layer was washed by dilute NaOH solution and then saturated NaCl solution, dried by $MgSO_4$ and filtered. Volatile organic solvent was evaporated under reduced pressure, and the crude product was purified by column chromatography on silica gel using hexane/ethyl acetate (10:1, v/v) as eluent, yielding **2a** as a white solid (2.02 g, 78%). ^1H NMR ($CDCl_3$, 400 MHz): δ = 6.93 (1H, t, J = 7.9 Hz), 6.29 (1H, d, J = 7.7 Hz), 6.11 (1H, d, J = 8.2 Hz), 4.50 (2H, s), 3.82 (1H, br), 3.52 (2H, t, J = 8.5 Hz), 3.04 (2H, t, J = 8.4 Hz), 1.48 (9H, s). $^{13}C\{^1H\}$ NMR ($CDCl_3$, 100 MHz) δ = 168.2, 154.7, 153.6, 128.3, 116.2, 103.6, 102.1, 81.9, 65.6, 47.3, 27.9, 26.8. MS (ESI$^+$): m/z 250 ($[M+H]^+$).

2b. The procedure was similar to that of **2a** except **1b** (4.2 g, 17.0 mmol) was used instead of **1a** and the other reagents were scaled up accordingly. The crude product was purified by column chromatography on silica gel using hexane/ethyl acetate (10:1, v/v) as eluent, yielding **2b** as a white solid (1.3 g, 30%). ^1H NMR ($CDCl_3$, 600 MHz): δ = 6.75 (1H, s), 6.58 (2H, s), 5.32 (1H, s), 4.41 (2H, s), 3.50 (2H, t, J =8.3 Hz), 2.97 (2H, t, J = 8.3 Hz), 1.47 (9H, s). $^{13}C\{^1H\}$ NMR ($CDCl_3$, 150 MHz) δ =168.6, 152.3, 145.4, 131.5, 113.4, 112.6, 110.6, 82.0, 66.9, 47.5, 30.3, 28.0.

3a. K_2CO_3 (2.24 g, 16.2 mmol) was added to **2a** (2.02 g, 8.1 mmol) in dry THF (40 mL). At 0 ^oC, acryloyl chloride (0.71 mL, 8.9 mmol) in dry THF (10 mL) was added dropwise to the solution mixture with vigorous stirring. The solution mixture was further stirred at 0 ^oC for 30 min, and then the reaction was quenched by addition of water. Any organic volatile was removed by evaporation under reduced pressure, and the aqueous layer was extracted with ethyl acetate. The ethyl acetate layer was then washed by saturated NaCl solution, dried by $MgSO_4$ and filtered. Volatile organic solvent was evaporated under reduced pressure, and the crude product was purified by column chromatography on silica gel using hexane/ethyl acetate (10:1, v/v) as eluent, yielding **3a** as a white solid (2.16 g, 88%). ^1H NMR ($CDCl_3$, 300 MHz): δ = 7.77 (1H, d, J = 7.7 Hz), 6.95 (1H, t, J = 8.2 Hz), 6.22-6.45 (3H, m), 5.58 (1H, dd, J = 2.9 and 9.3 Hz), 4.36 (2H, s), 3.89-3.99 (2H, m), 2.96 (2H, t, J = 8.3 Hz), 1.32 (9H, s). $^{13}C\{^1H\}$ NMR ($CDCl_3$, 75 MHz) δ = 170.5, 167.5, 163.3, 153.8, 144.1, 128.9, 128.4, 119.2, 110.7, 106.7, 81.7, 65.2, 48.1, 27.6, 24.6. MS (ESI$^+$): m/z 304 ($[M+H]^+$).

3b. The procedure was similar to that of **3a** except that **2b** (1.1 g, 4.4 mmol) was used instead of **2a** and the other reagents were scaled down accordingly. The crude product was purified by column chromatography on silica gel using hexane/ethyl acetate (10:1, v/v) as eluent, yielding **3b** as a white solid (1.1 g, 82%). ^{1}H NMR ($CDCl_3$, 500 MHz): δ = 8.21 (1H, d, J = 8.80 Hz), 6.71-6.78 (2H, m), 6.46-70 (2H, m), 5.77 (1H, dd, J = 2.00 and 9.78 Hz), 4.49 (2H, s), 4.16 (2H, t, J=8.35), 3.17 (2H, t, J = 8.35 Hz), 1.48 (9H, s). MS (ESI$^+$): m/z 304 ([M+H]$^+$).

4a. 2,3-Dichloro-5,6-dicyano-*p*-benzoquinone (DDQ; 603 mg, 2.7 mmol) was added to **3a** (2.0 mmol) in dry toluene (15 mL), and the solution mixture was heated to reflux with vigorous stirring overnight. The reaction mixture was then diluted by ethyl acetate and washed by water and saturated NaCl solution. The organic layer was dried by $MgSO_4$, filtered and evaporated under reduced pressure. The crude product was purified by column chromatography on silica gel using hexane/ethyl acetate (10:1, v/v) as eluent, yielding **4a** as a white solid (560 mg, 93%). ^{1}H NMR ($CDCl_3$, 400 MHz): δ = 8.13 (1H, d, J = 8.3 Hz), 7.41 (1H, d, J = 3.8 Hz), 7.24 (1H, t, J = 8.1 Hz), 6.84-6.98 (2H, m), 6.57-6.68 (2H, m), 5.99 (1H, d, J = 10.9 Hz), 4.64 (2H, s), 1.48 (9H, s). ^{13}C{^{1}H} NMR ($CDCl_3$, 100 MHz) δ = 168.0, 164.0, 151.1, 137.2, 132.1, 127.9, 125.9, 123.4, 121.1, 110.7, 106.5, 105.5, 82.4, 66.0, 28.1. MS (ESI$^+$): m/z 324 ([M+Na]$^+$).

4b. The procedure was similar to that of **4a** except that **3b** (1 g, 3.31 mmol) was used instead of **3a** and the other reagents were scaled up accordingly. The crude product was purified by column chromatography on silica gel using hexane/ethyl acetate (10:1, v/v) as eluent, yielding **4b** as a white solid (626.1 mg, 63%). ^{1}H NMR ($CDCl_3$, 600 MHz): δ = 8.42 (1H, d, J = 8.94 Hz), 7.50 (1H, d, J = 3.78 Hz), 7.03 (1H, d, J = 2.46 Hz), 6.99-7.01 (1H, m), 6.93-6.97 (1H, m), 6.67 (1H, dd, J = 1.32 and 16.77 Hz), 6.60 (1H, d, J = 3.6 Hz), 6.03 (1H, dd, J = 1.38 and 10.44 Hz), 4.57 (2H, s), 1.49 (9H, s). MS (ESI$^+$): m/z 324 ([M+Na]$^+$).

5a. 85% H_3PO_4 (1 mL) was added to **4a** (100 mg, 0.33 mmol) in MeCN (1 mL). The solution mixture was stirred at room temperature overnight. The reaction was then quenched by the addition of water, and organic volatile was removed by evaporation under reduced pressure. The aqueous layer was extracted with ethyl acetate twice and the combined ethyl acetate fraction was dried by $MgSO_4$ and filtered. Volatile organic solvent was evaporated under reduced pressure, and the crude product was purified by column chromatography on silica gel using dichloromethane/methanol (10:1, v/v) as eluent, yielding **5a** as a white solid (80 mg, 98%). ^{1}H NMR (CD_3O_D, 400 MHz): δ = 8.08 (1H, d, J = 8.3 Hz), 7.72 (1H, d, J = 3.9 Hz),

7.16-7.27 (2H, m), 6.90 (1H, d, *J* = 3.9 Hz), 6.73 (1H, d, *J* = 8.0 Hz), 6.62 (1H, dd, *J* = 1.6 and 15.9 Hz), 6.06 (1H, dd, *J* = 1.6 and 10.3 Hz), 4.79 (2H, s). MS (ESI$^-$): *m/z* 244 ([M–H]$^-$).

5b. The procedure was similar to that of **5a** except that **4b** (84.6 mg, 0.28 mmol) was used instead of **4a** and the other reagents were scaled down accordingly. The crude product was purified by column chromatography on silica gel using dichloromethane/methanol (10:1, v/v) as eluent, yielding **5b** as a white solid (48.5 mg, 70%). ^{1}H NMR (CD_3O_D, 600 MHz): δ = 8.26 (1H, d, *J* = 9.06 Hz), 7.71 (1H, d, *J* = 3.78 Hz), 7.09-7.13 (1H, m), 7.02 (1H, d, *J* = 2.58 Hz), 6.89-6.91 (1H, m), 6.50-6.57 (2H, m), 5.96 (1H, dd, *J* = 1.62 and 10.4 Hz), 4.60 (2H, s). MS (ESI$^-$): *m/z* 244 ([M–H]$^-$).

NAIA-4. HATU (48.2 mg, 0.13 mmol) was added to **5a** (30 mg, 0.12 mmol) in dry DMF (2 mL) and the reaction mixture was stirred at room temperature for 15 min. After that, hex-5-yn-1-amine (12.8 µL, 0.11 mmol) in dry DMF (1 mL) was added to the solution mixture, followed by the addition of DIPEA (57.8 µL, 0.33 mmol). The solution was stirred at room temperature overnight. The reaction was then quenched by the addition of water. Any organic volatile was removed by evaporation under reduced pressure, and the aqueous layer was extracted with ethyl acetate twice. The combined ethyl acetate fraction was washed by saturated NaCl solution, dried by $MgSO_4$ and filtered. Volatile organic solvent was evaporated under reduced pressure, and the crude product was purified by column chromatography on silica gel using dichloromethane/methanol (20:1, v/v) as eluent, yielding NAIA-4 as a white solid (25 mg, 70%). ^{1}H NMR ($CDCl_3$, 400 MHz): δ = 8.18 (1H, d, *J* = 8.4 Hz), 7.49 (1H, d, *J* = 3.8 Hz), 7.30 (1H, t, *J* = 8.2 Hz), 6.98 (1H, dd, *J* = 10.4 and 16.0 Hz), 6.80 (1H, d, *J* = 3.8 Hz), 6.67-6.74 (2H, m), 6.61 (1H, br), 6.08 (1H, dd, *J* = 1.4 and 10.4 Hz), 4.65 (2H, s), 3.40 (2H, q, *J* = 6.8 Hz), 2.22 (2H, dt, *J* = 2.7 and 6.8 Hz), 1.95 (2H, t, *J* = 2.6 Hz), 1.64-1.73 (2H, m), 1.50-1.59 (2H, m). ^{13}C{^{1}H} NMR ($CDCl_3$, 100 MHz) δ = 168.3, 164.1, 150.4, 137.3, 132.6, 127.9, 126.3, 123.9, 120.9, 111.4, 106.2, 105.8, 84.0, 68.9, 68.0, 38.6, 28.7, 25.7, 18.2. HRMS (ESI) m/z [M+Na]$^+$ calcd for $C_{19}H_{20}N_2O_3Na$, 347.1366; found, 347.1367.

NAIA-5. The procedure was similar to that of NAIA-4 except that **5b** (50.9 mg, 0.21 mmol) was used instead of **5a** and the other reagents were scaled up accordingly. The crude product was purified by column chromatography on silica gel using dichloromethane/methanol (20:1, v/v) as eluent, yielding NAIA-5 as a white solid (24.9 mg, 37%). ^{1}H NMR ($CDCl_3$, 600 MHz): δ = 8.45 (1H, d, *J* = 9.00 Hz), 7.53 (1H, d, *J* = 3.72 Hz), 7.05 (1H, d, *J* = 2.58 Hz), 7.01 (1H,

dd, J = 2.52 and 9.00 Hz), 6.96 (1H, dd, J = 10.4 and 16.7 Hz), 6.68 (2H, dd, J = 1.32 and 16.7 Hz), 6.62 (1H, d, J=3.72), 6.05 (1H, dd, J- 1.32 and 5.25 Hz), 4.25 (2H, s), 3.39 (2H, q, J = 6.9 Hz), 2.22 (2H, td, J = 2.58 and 6.94 Hz), 1.95 (1H, t, J = 2.64 Hz), 1.66-1.71 (2H, m), 1.54-1.58 (2H, m). $^{13}C\{^1H\}$ NMR ($CDCl_3$, 150 MHz) δ = 168.3, 163.6, 154.2, 132.2, 131.7, 131.2, 127.6, 125.6, 118.0, 113.9, 109.2, 104.7, 83.9, 38.5, 28.6, 25.6, 18.1. MS (ESI^+): *m/z* 325 ($[M+H]^+$).

Supplementary Table 1. Manders's colocalization coefficient of the TAMRA channel with the Hoechst channel in the NAIA- and IAA-treated cells; *n* = 10 cells from 3 different images

Sample	Mander's colocalization coeffficient
Cells treated with IAA for 15 min	0.52±0.06
Cells treated with IAA for 30 min	0.60±0.02
Cells treated with IAA for 60 min	0.63±0.03
Cells treated with NAIA for 15 min	0.59±0.05
Cells treated with NAIA for 30 min	0.65±0.04
Cells treated with NAIA for 60 min	0.70±0.05

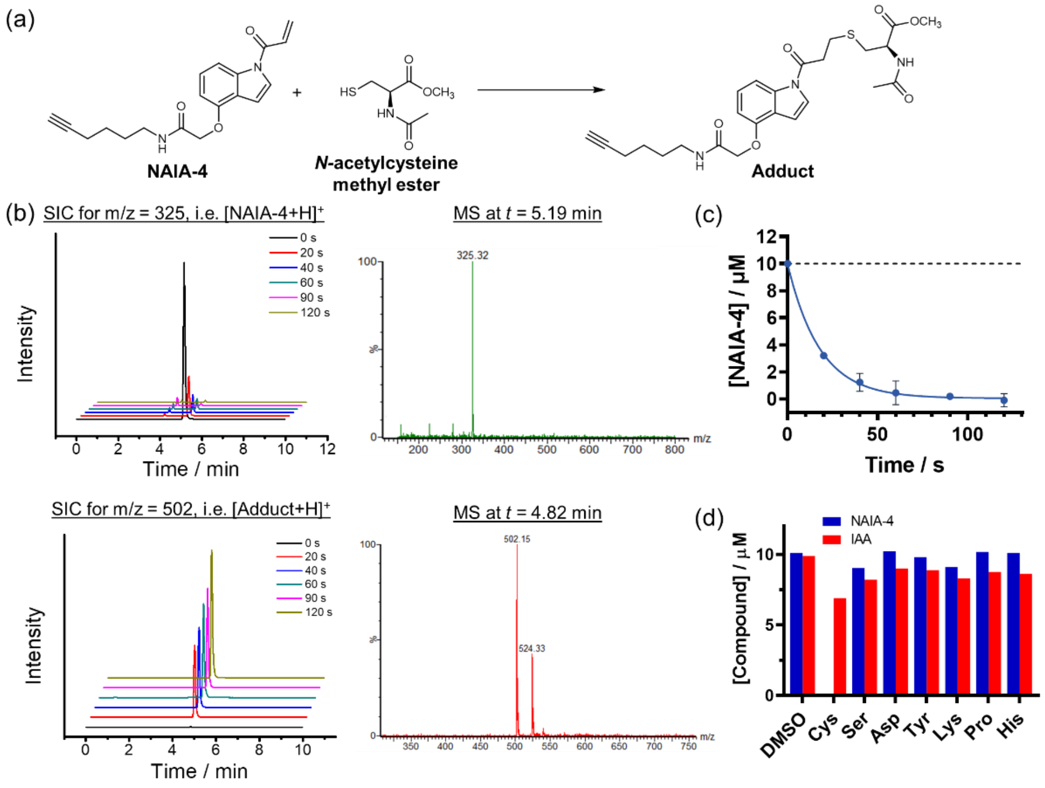

Supplementary Figure 1. LC-MS analysis of the reaction between NAIA-4 and small amino acids in aqueous buffer solution. (a) Chemical equation showing thiol-Michael addition of NAIA-4 with *N*-acetylcysteine methyl ester (*N*-Ac-Cys-OMe), a N- and C-terminal protected cysteine, to form the adduct product. (b) NAIA-4 (10 µM) in aqueous buffer solution (PBS-MeOH, 4:1, v/v) was incubated with *N*-Ac-Cys-OMe (250 µM). At indicated time intervals, an aliquot of the solution mixture was sent for LC-MS analysis. Selected ion chromatograms (SIC) at m/z = 325 and 502, corresponding to the molecular ion of $[NAIA\text{-}4+H]^+$ and $[Adduct+H]^+$ respectively, show a consumption of NAIA-4 with concomitant formation of the Cys adduct. The mass spectra (MS) at 5.19 and 4.82 min confirm the identity of NAIA-4 and adduct. (c) Changes in NAIA-4 level over time upon incubation with *N*-Ac-Cys-OMe (250 µM) in aqueous buffer solution (PBS-MeOH, 4:1, v/v). Quantified data were shown in average±SD from n = 4 replicates/group. (d) Selectivity of NAIA-4 toward reaction with Cys over other nucleophilic amino acids, as compared to IAA.

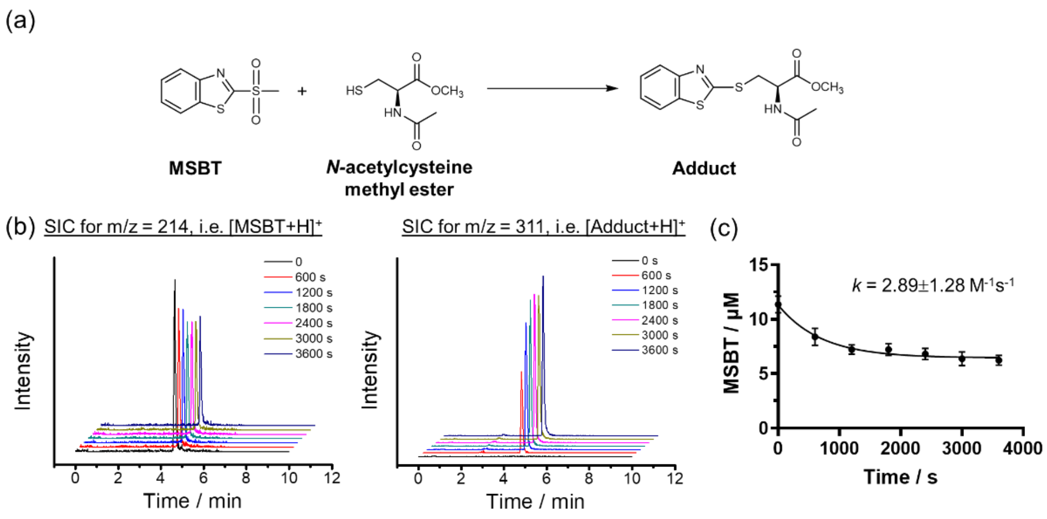

Supplementary Figure 2. LC-MS analysis of the reaction between methylsulfonylbenzothiazole (MSBT) and *N*-Ac-Cys-OMe in aqueous buffer solution. (a) Chemical equation showing nucleophilic aromatic substitution reaction of MSBT with *N*-acetylcysteine methyl ester (*N*-Ac-Cys-OMe), a N- and C-terminal protected cysteine, to form the adduct product. (b) MSBT (11.4 µM) in aqueous buffer solution (PBS-MeOH, 4:1, v/v) was incubated with *N*-Ac-Cys-OMe (250 µM). At indicated time intervals, an aliquot of the solution mixture was sent for LC-MS analysis. Selected ion chromatograms (SIC) at m/z = 214 and 311, corresponding to the molecular ion of $[MSBT+H]^+$ and $[Adduct+H]^+$ respectively. (c) Changes in MBST level over time upon incubation with *N*-Ac-Cys-OMe (250 µM) in the aqueous buffer solution. Quantified data were shown in average±SD from *n* = 3 replicates/group. Biomolecular reaction rate constant was determined as 2.89±1.28 $M^{-1}s^{-1}$

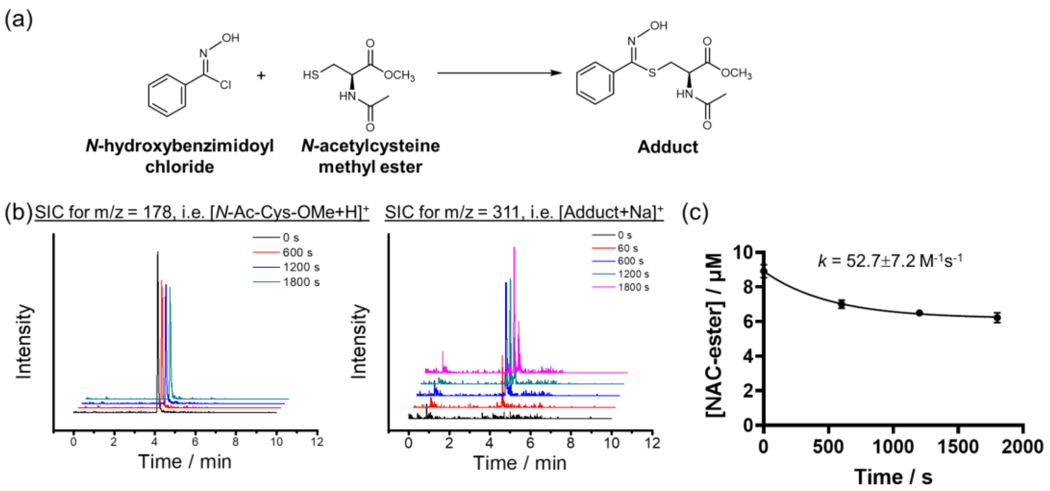

Supplementary Figure 3. LC-MS analysis of the reaction between *N*-hydroxybenzimidoyl chloride and *N*-Ac-Cys-OMe in aqueous buffer solution. (a) Chemical equation showing nucleophilic substitution reaction of *N*-hydroxybenzimidoyl chloride with *N*-acetylcysteine methyl ester (*N*-Ac-Cys-OMe) to form the adduct product. (b) *N*-hydroxybenzimidoyl chloride (10 µM) in aqueous buffer solution (PBS-MeOH, 4:1, v/v) was incubated with *N*-Ac-Cys-OMe (10 µM). At indicated time intervals, an aliquot of the solution mixture was sent for LC-MS analysis. Selected ion chromatograms (SIC) at m/z = 178 and 311, corresponding to the molecular ion of $[N\text{-Ac-Cys-OMe+H}]^+$ and $[\text{Adduct+Na}]^+$ respectively. Note that the molecular ion of *N*-hydroxybenzimidoyl chloride cannot be detected by the ESI^+ MS. (c) Changes in *N*-Ac-Cys-OMe level over time upon incubation with *N*-hydroxybenzimidoyl chloride (10 µM) in the aqueous buffer solution. Quantified data were shown in average±SD from n = 2 replicates/group. Biomolecular reaction rate constant was determined as 52.7±7.2 $M^{-1}s^{-1}$

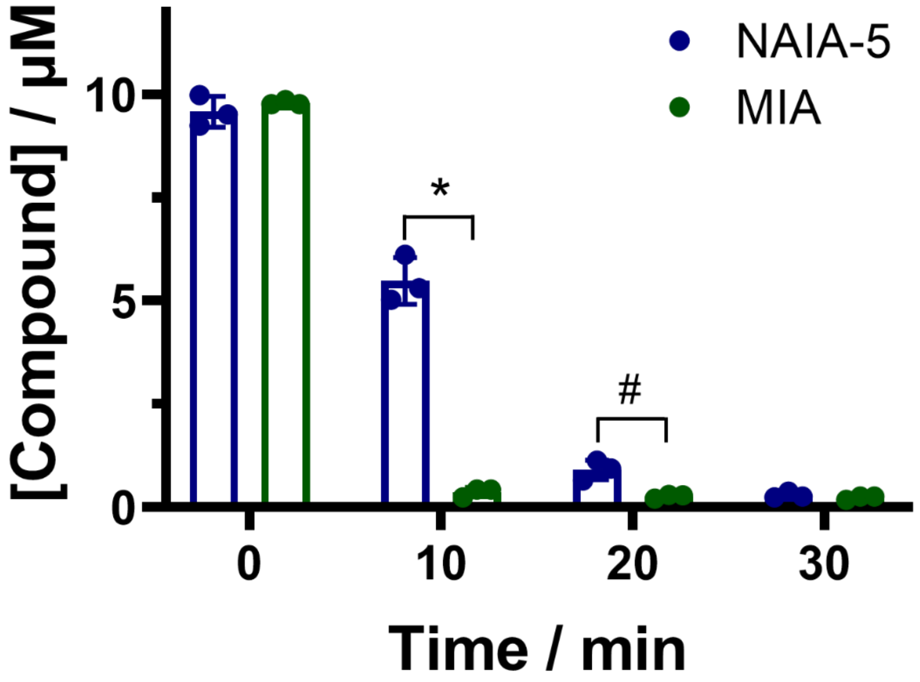

Supplementary Figure 4. LC-MS analysis of the reaction of NAIA-5 and MIA, respectively, with glutathione (GSH) in aqueous buffer solution. NAIA-5 or **MIA** (10 μM) in aqueous buffer solution (PBS-MeOH, 4:1, v/v) was incubated with GSH (30 μM). At indicated time intervals, an aliquot of the solution mixture was sent for LC-MS analysis. Quantified data were shown in average±SD from n = 3 different replicates/group. Statistical analyses were performed with unpaired two-tailed Student's t-tests. Statistical significance is expressed as $^{*}P=1.03\times10^{-4}$ and $^{\#}P=1.05\times10^{-2}$ respectively.

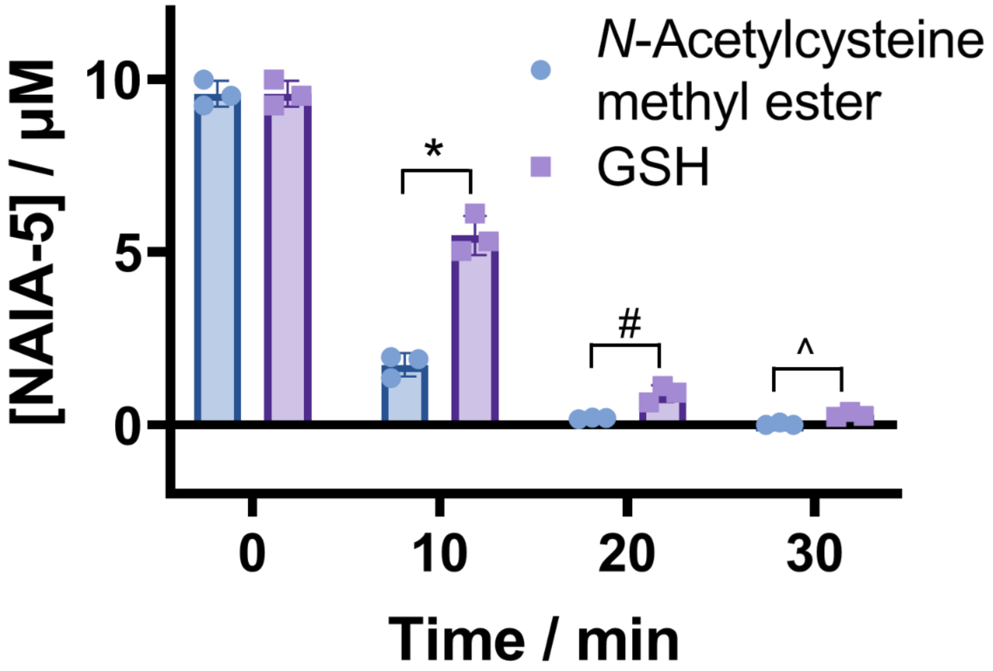

Supplementary Figure 5. LC-MS analysis of reactions of NAIA-5 with *N*-acetylcysteine methyl ester and GSH. NAIA-5 (10 µM) in aqueous buffer solution (PBS-MeOH, 4:1, v/v) was incubated with *N*-acetylcysteine methyl ester or GSH (30 µM). At indicated time intervals, an aliquot of the solution mixture was sent for LC-MS analysis. Quantified data were shown in average±SD from n = 3 different replicates/group. Statistical analyses were performed with unpaired two-tailed Student's t-tests. Statistical significance is expressed as $^{*}P=5.88\times10^{-4}$, $^{\#}P=6.93\times10^{-3}$ and $^{\wedge}P=5.20\times10^{-3}$ respectively.

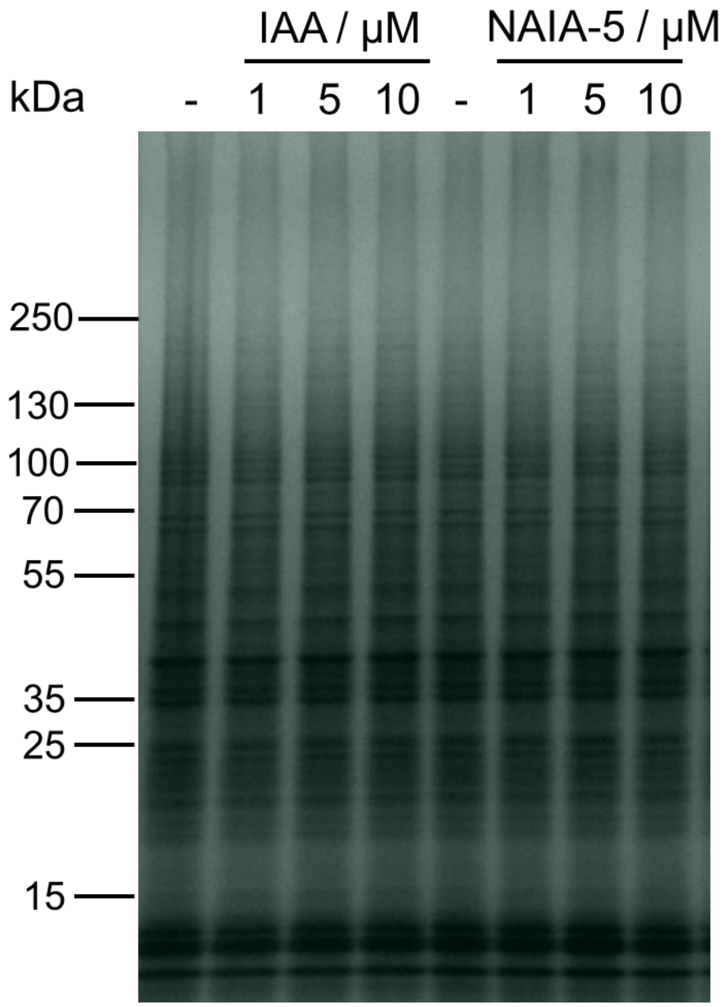

Supplementary Figure 6. SimplyBlue™ SafeStain of gel in Figure 3a. No significant change in band intensity was found, indicating the difference in in-gel fluorescence intensity in Figure 3a originates from the different extents of cysteine labeling by NAIA-5 and IAA instead of different protein loadings. Similar results were found in 3 replicate experiments.

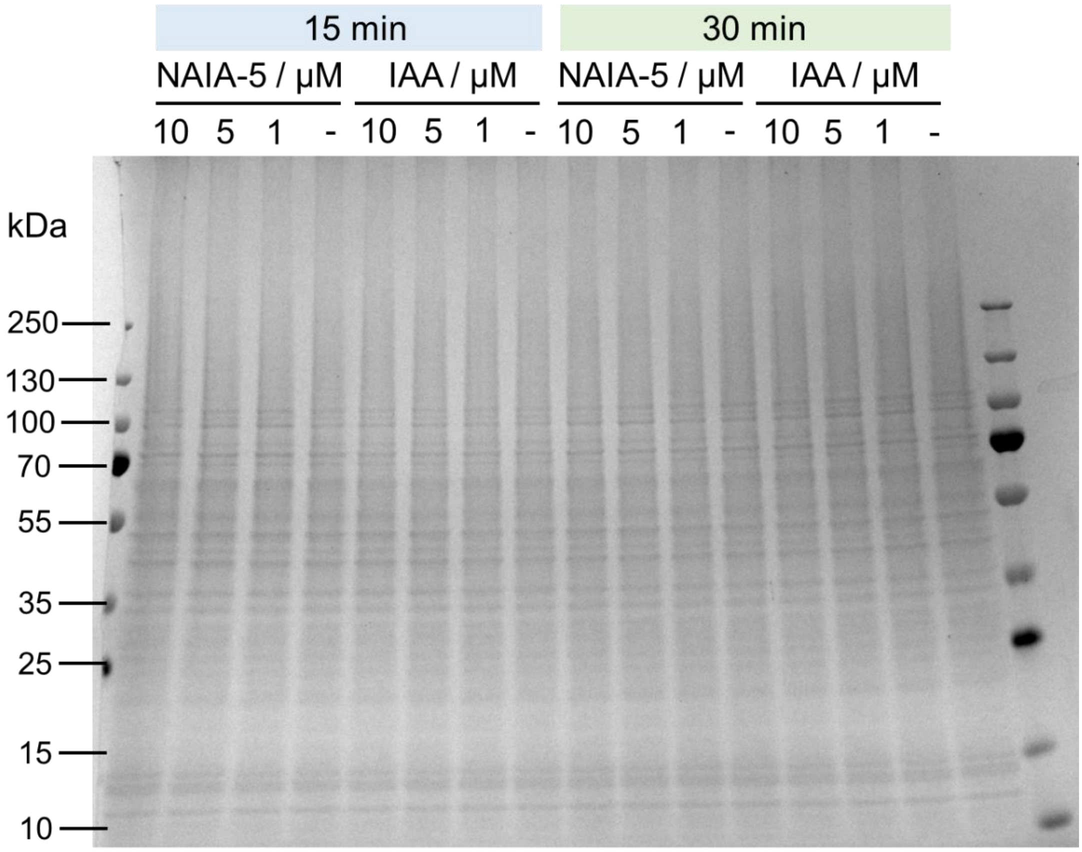

Supplementary Figure 7. SimplyBlue™ SafeStain of gel in Figure 3b. No significant change in band intensity was found, indicating the difference in in-gel fluorescence intensity in Figure 3b originates from the different extents of cysteine labeling by NAIA-5 and IAA instead of different protein loadings. Result from a single experiment.

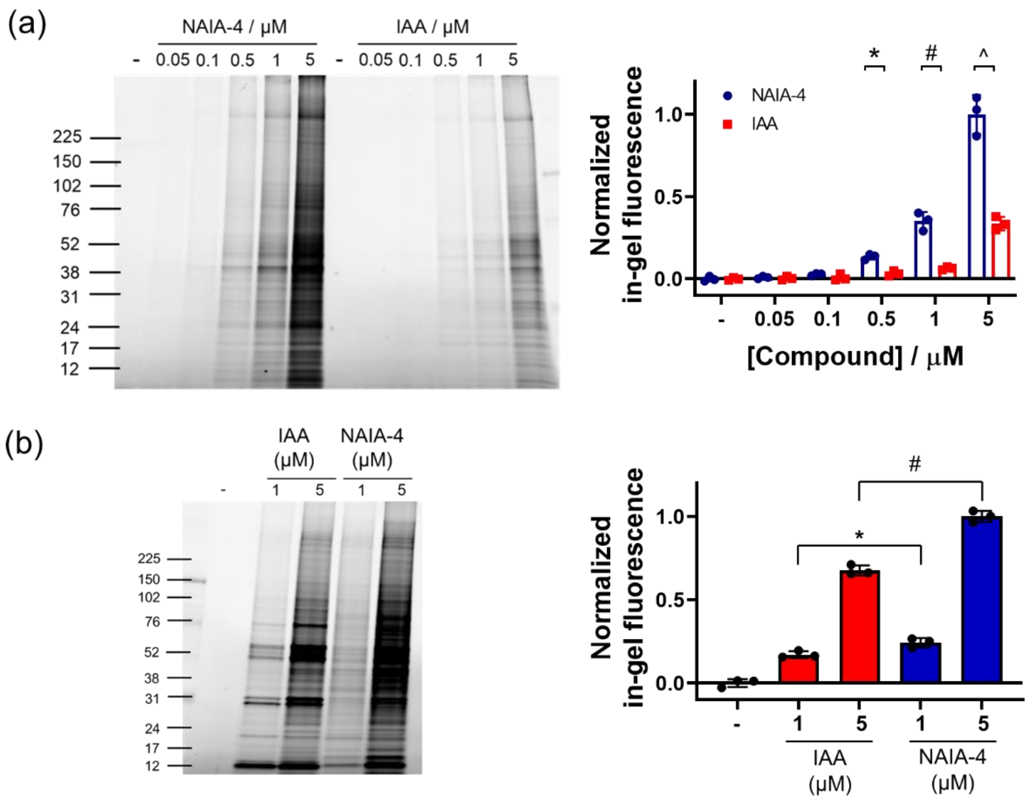

Supplementary Figure 8. Gel-based ABPP experiments of NAIA-4 to investigate its ability to label Cys in cell lysates and live cells. (a) 231MFP cell lysates (20 μg) were incubated with NAIA-4 or IAA at indicated concentrations for 30 min, following by CuAAC reaction with azide-fluor 545 (5 μM). The labeled proteins were then boiled with sampling buffer, and read out by in-gel fluorescence after SDS-PAGE. Quantified data were shown in average±s.e.m. from n = 3 replicates/group. Statistical significance *vs* untreated control is expressed as $^{*}p < 0.05$; $^{***}p < 0.001$, and *vs* different treatment groups is expressed as $^{\#\#}p < 0.01$; $^{\#\#\#}p < 0.001$. (b) 231MFP cells were pre-treated with NAIA-4 or IAA at indicated concentrations for 1h. The cells were then washed with DPBS and lysed in DPBS by probe sonication. The cell lysates were labeled with azide-fluor 545 (25 μM) by CuAAC reaction, and the labeled protein were detected by in-gel fluorescence after SDS-PAGE. Quantified data were shown in average±s.e.m. from n = 3 biological replicates/group. Statistical analyses were performed with unpaired two-tailed Student's t-tests. Statistical significance is expressed as $^{*}P=1.64\times10^{-3}$, $^{\#}P=8.73\times10^{-4}$ and $^{\wedge}P=7.94\times10^{-4}$ respectively in (a); $^{*}P=2.29\times10^{-2}$ and $^{\#}P=2.38\times10^{-4}$ respectively in (b).

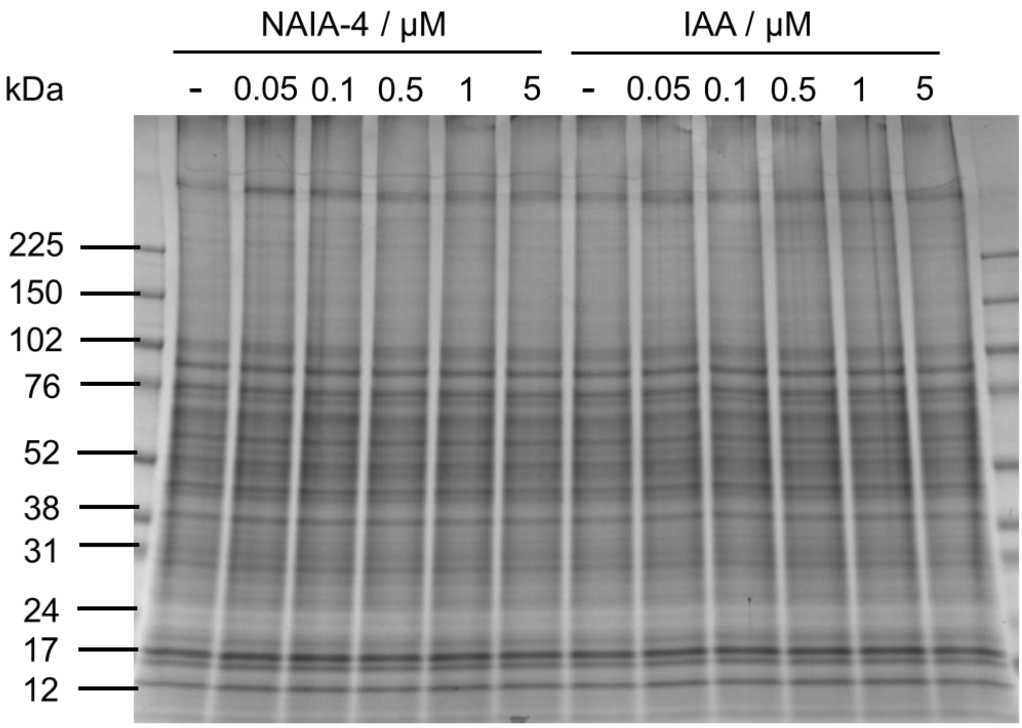

Supplementary Figure 9. SimplyBlue™ SafeStain of gel in Supplementary Figure 8a. No significant change in band intensity was found, indicating the difference in in-gel fluorescence intensity in Supplementary Figure 8a originates from the different extents of cysteine labeling by NAIA-4 and IAA instead of different protein loadings. Similar results were found in 3 replicate experiments.

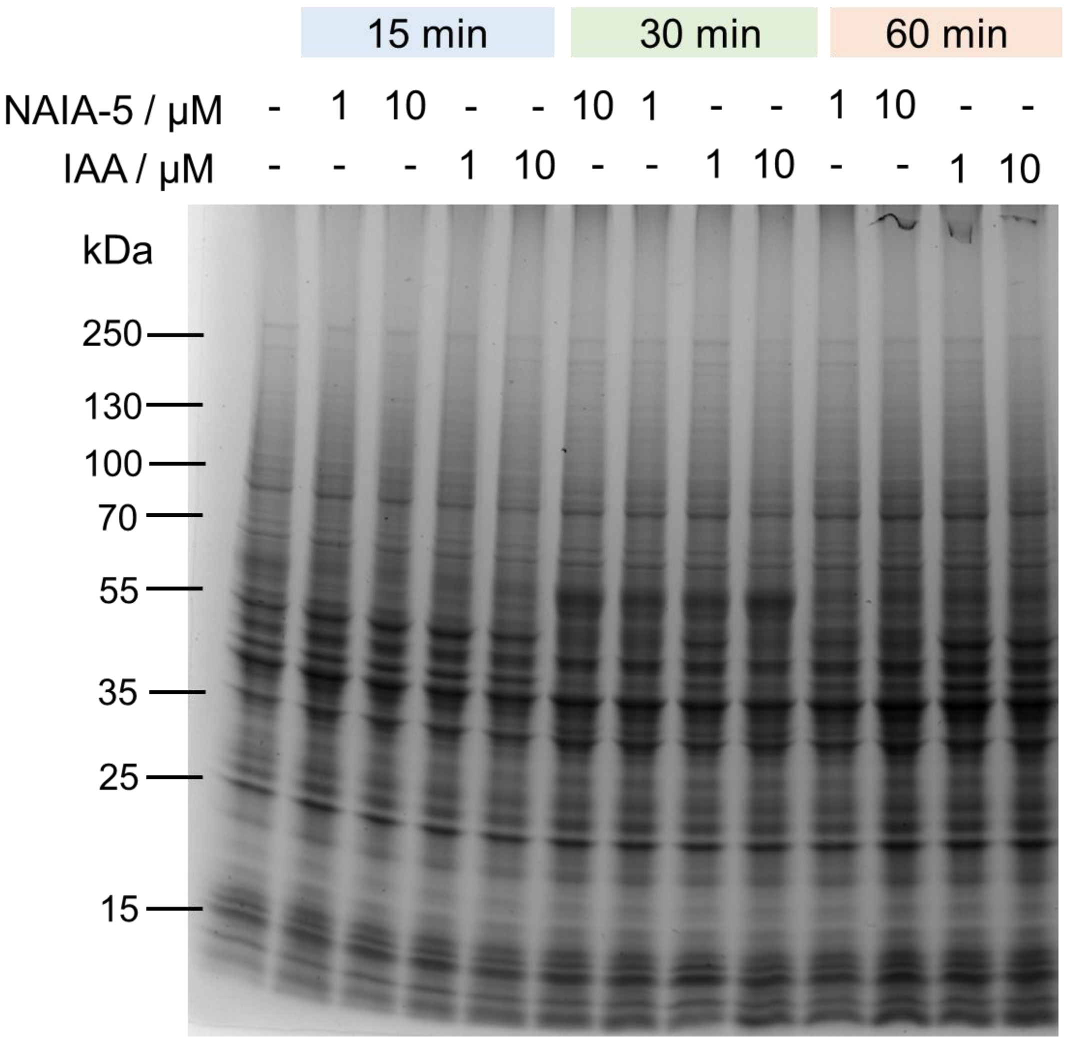

Supplementary Figure 10. SimplyBlue™ SafeStain of gel in Figure 3d. No significant change in band intensity was found, indicating the difference in in-gel fluorescence intensity in Figure 3d originates from the different extents of cysteine labeling by NAIA-5 and IAA instead of different protein loadings. Similar results were found in 2 replicate experiments.

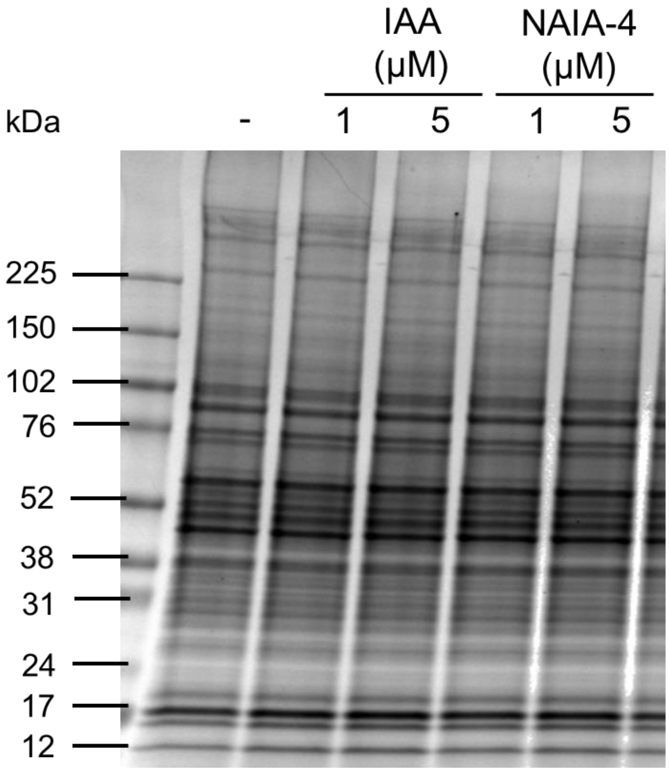

Supplementary Figure 11. SimplyBlue™ SafeStain of gel in Supplementary Figure 8b. No significant change in band intensity was found, indicating the difference in in-gel fluorescence intensity in Supplementary Figure 8b originates from the different extents of cysteine labeling by NAIA-4 and IAA instead of different protein loadings. Similar results were found in 3 replicate experiments.

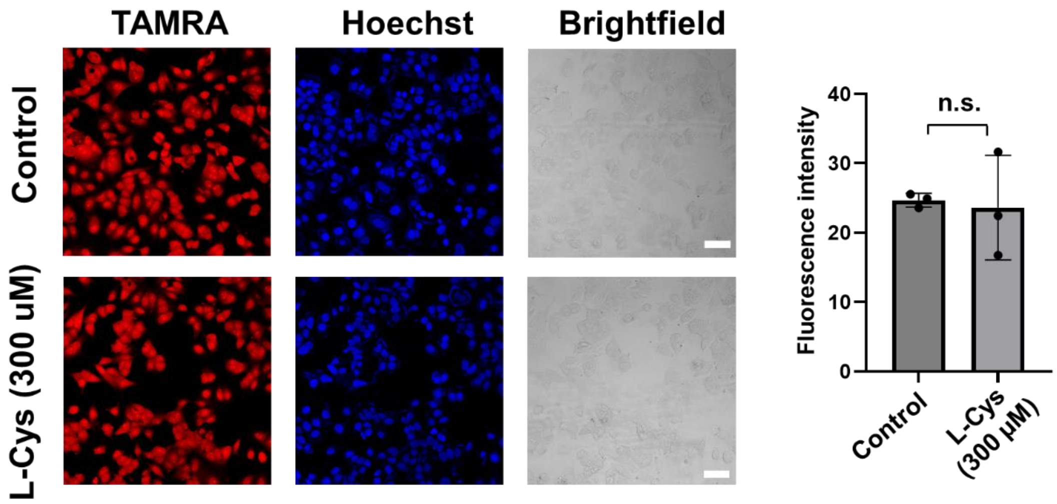

Supplementary Figure 12. Imaging free thiols in HepG2 cells with elevated GSH levels. HepG2 cells on 8-well chambered slide were pre-treated with solvent vehicle or L-Cys (300 μM) in complete medium for 16 h, where the latter is to stimulate endogenous glutathione synthesis. The cells were washed by PBS, and incubated with NAIA-5 (10 μM) in complete medium for 30 min. The cells were then washed with PBS, fixed by 4% paraformaldehyde, permeabilized and reacted with azide-fluor 545 (20 μM) by CuAAC reaction at room temperature in dark for 1 h. The stained cells were washed with PBS and further incubated with Hoechst (8.2 μM) for 15 min. The cells were then washed with PBS and imaged by confocal fluorescence microscopy. Cellular fluorescence intensity was determined by ImageJ. Quantified data were shown in average±SD from n = 3 different biological replicates/group. Statistical analyses were performed with unpaired two-tailed Student's t-tests. n.s. = not significant. Scale bar = 50 μm

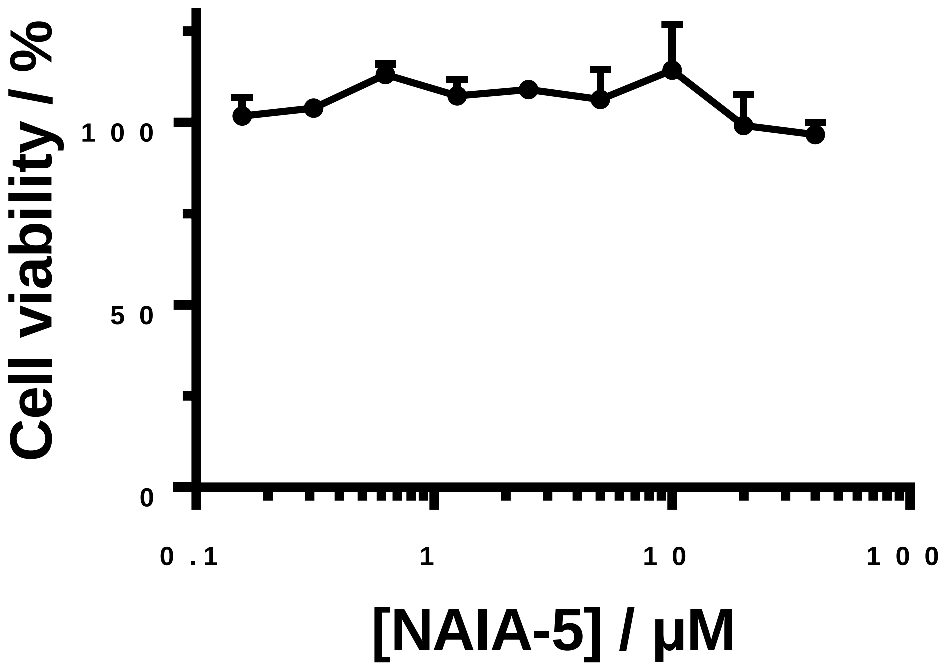

Supplementary Figure 13. MTT assay reveals no significant changes in viability of HepG2 cells after treatment with NAIA-5 for 1 h. Quantified data were shown in average±SD from *n* = 3 biological replicates/group.

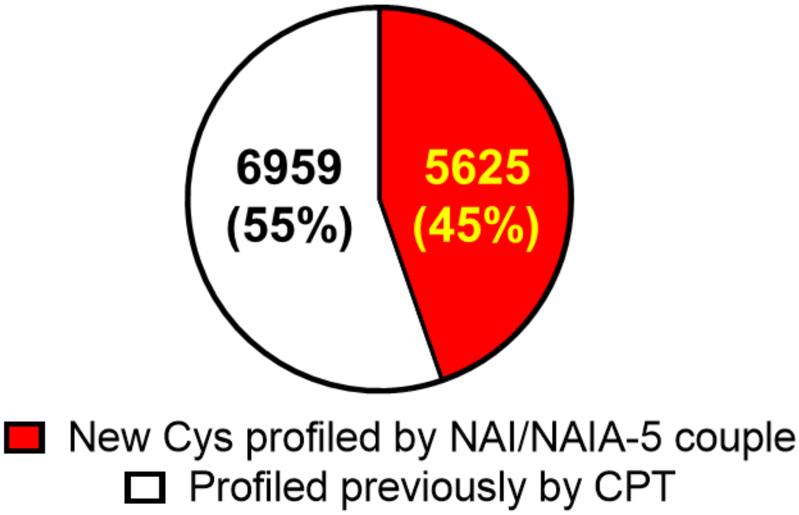

Supplementary Figure 14. Comparison of the Cys identified by NAI/NAIA-5 couple in HepG2 cells with the reported results from redox proteomics using cysteine-reactive phosphate tag (CPT; *Cell* **180**, 968-983.e24 (2020).

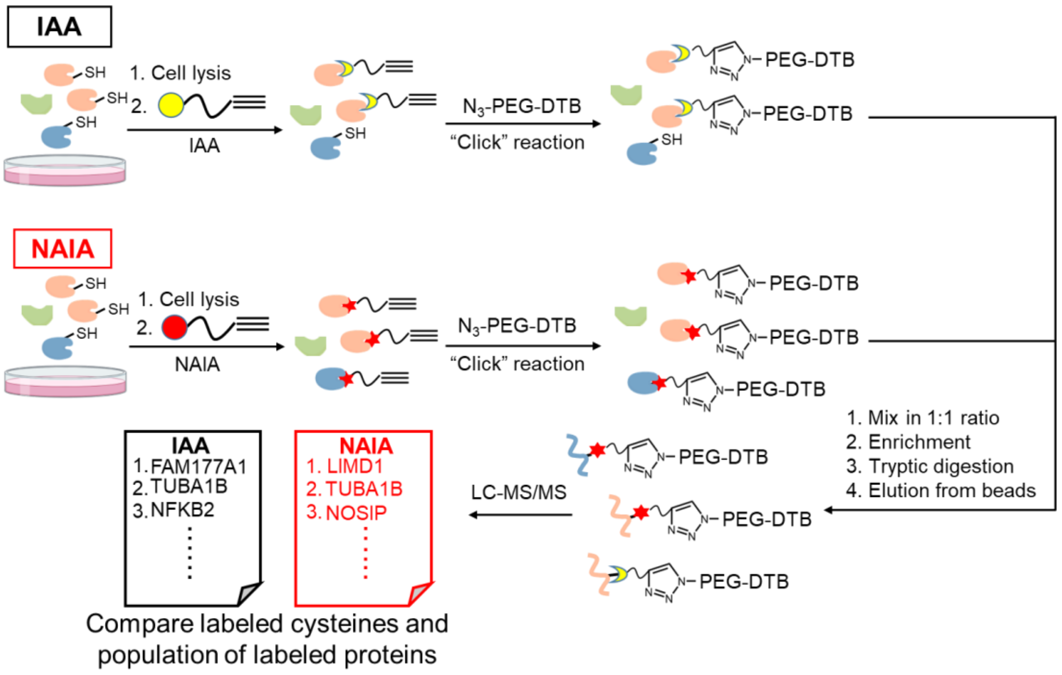

Supplementary Figure 15. Schematic cartoon illustrating workflow of MS-based ABPP experiment to compare cysteine profiling by NAIA vs IAA. For cell lysate labeling experiment, cells in PBS were lysed by sonication and cell debris were removed by centrifugation. After protein assay and normalization, the cell lysates were incubated with NAIA-5 (10 µM) or IAA (10 µM) at room temperature for 1 h, followed by installation of desthiobiotin (DTB) onto the labeled proteins by CuAAC reaction with DTB-PEG-azide. Then NAIA-5 and IAA treated samples were mixed in 1:1 ratio, and subjected to streptavidin bead pull-down, cysteine carbamidomethylation, on-bead tryptic digestion and peptide elution from the bead using acetonitrile-water mixture (1:1, v/v; with 0.1% formic acid). The eluted peptides were sent for LC-MS/MS analysis. The MS data were then searched for peptides with NAIA-5 and IAA specific modifications on Cys by MaxQuant. For live-cell labeling experiment, the cells were incubated with NAIA-5 (10 µM) or IAA (10 µM) in complete media for 1 h, washed with PBS and lysed by sonication. Then the samples were subjected to the same preparation procedure as the cell lysate labeling experiment. Illustration was created using ChemDraw, Biorender.com and MS Powerpoint.

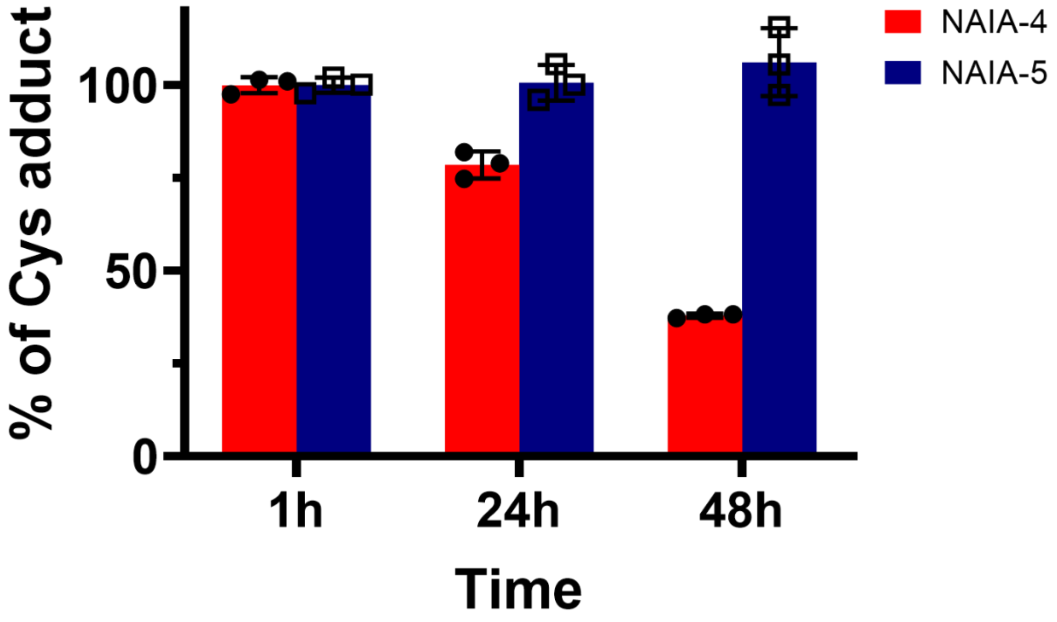

Supplementary Figure 16. LC-MS analysis of stability of NAIA-Cys adduct in aqueous buffer solution. NAIA-4 or **NAIA-5** (10 μM) in aqueous buffer solution (PBS-MeOH, 4:1, v/v) was incubated with *N*-Ac-Cys-OMe (30 μM) at room temperature. At indicated time intervals, an aliquot of the solution mixture was sent for LC-MS analysis on the level of NAIA-Cys adduct in the solution mixture. Quantified data were shown in average±SD from n = 3 replicates/group.

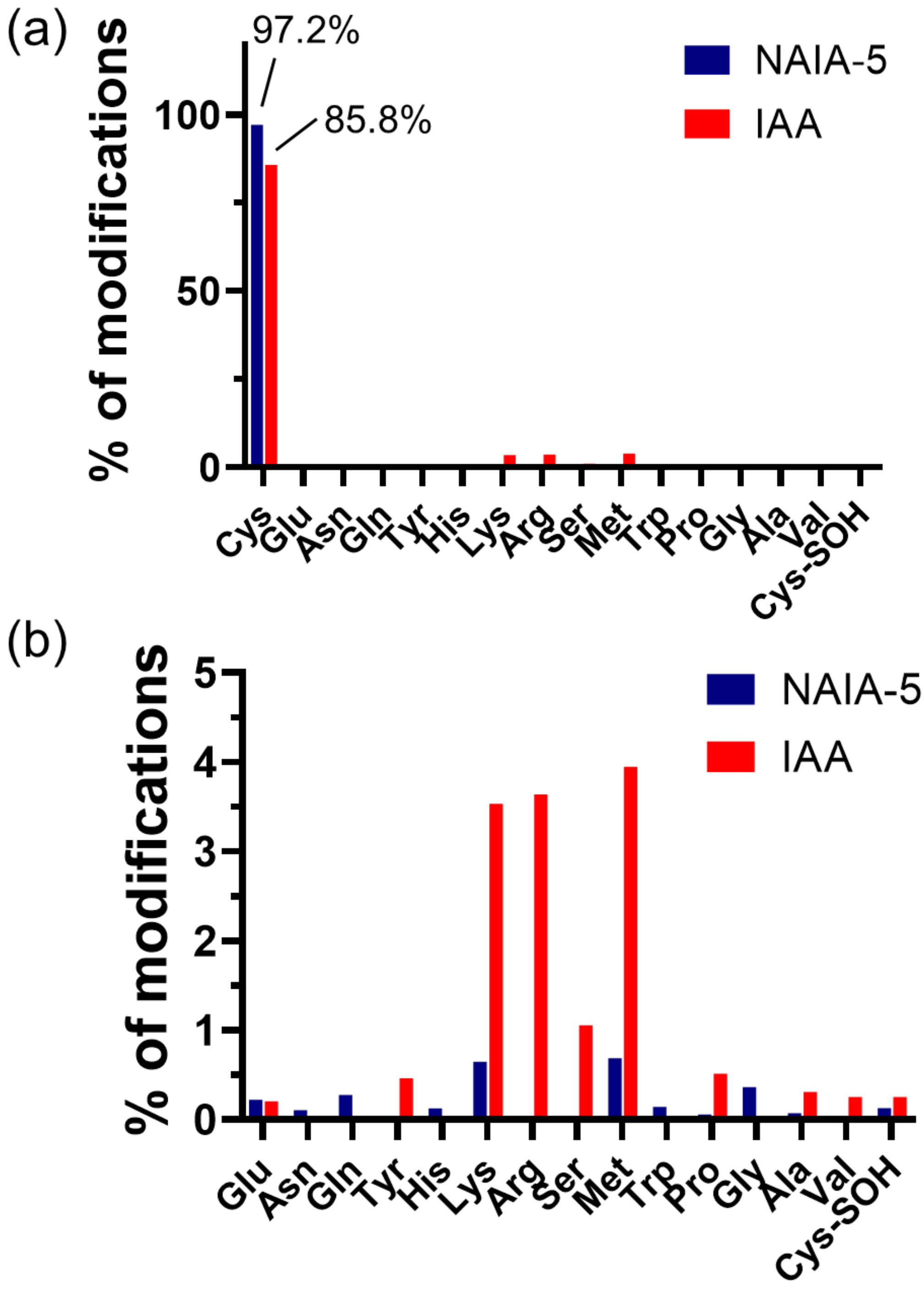

Supplementary Figure 17. (a) Percentage of probe modifications on different amino acids in HepG2 cell lysates labeled by NAIA-5 and IAA respectively. Cys-SOH: sulfenic acid form of Cys. (b) A plot without Cys for better visualization of the labeling on other amino acids.

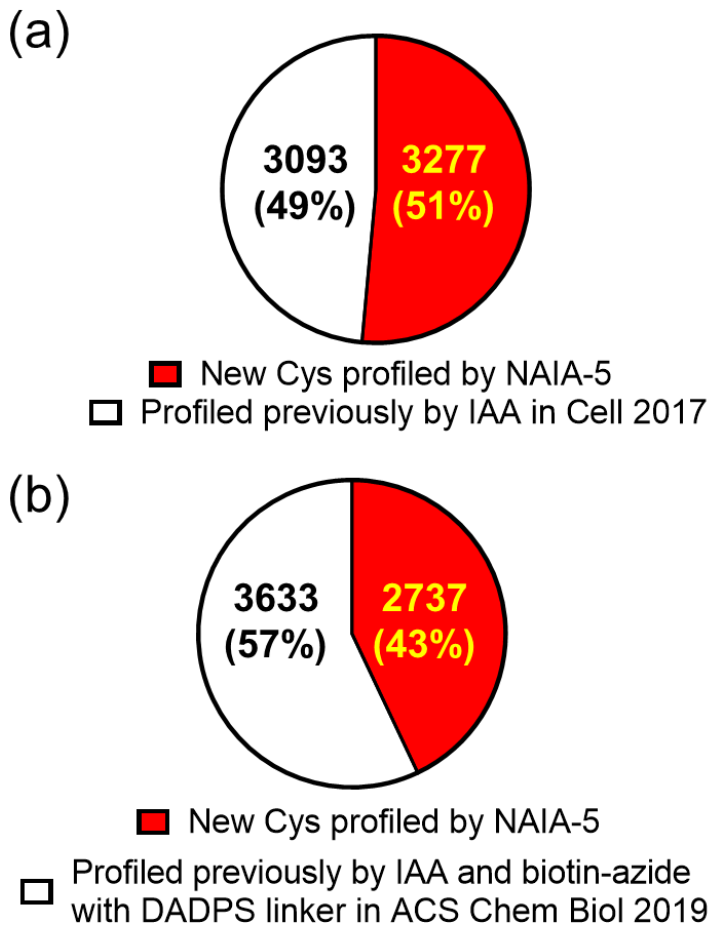

Supplementary Figure 18. Comparison of functional cysteines in HepG2 cell lysates identified by NAIA-5 with the results from reported cysteine profiling experiments using (a) IAA under optimal conditions (*Cell* **171**, 696-709.e23 (2017)), or (b) IAA and biotin-azide with chemically cleavable linker (*ACS Chem. Biol.* **14**, 1940–1950 (2019)).

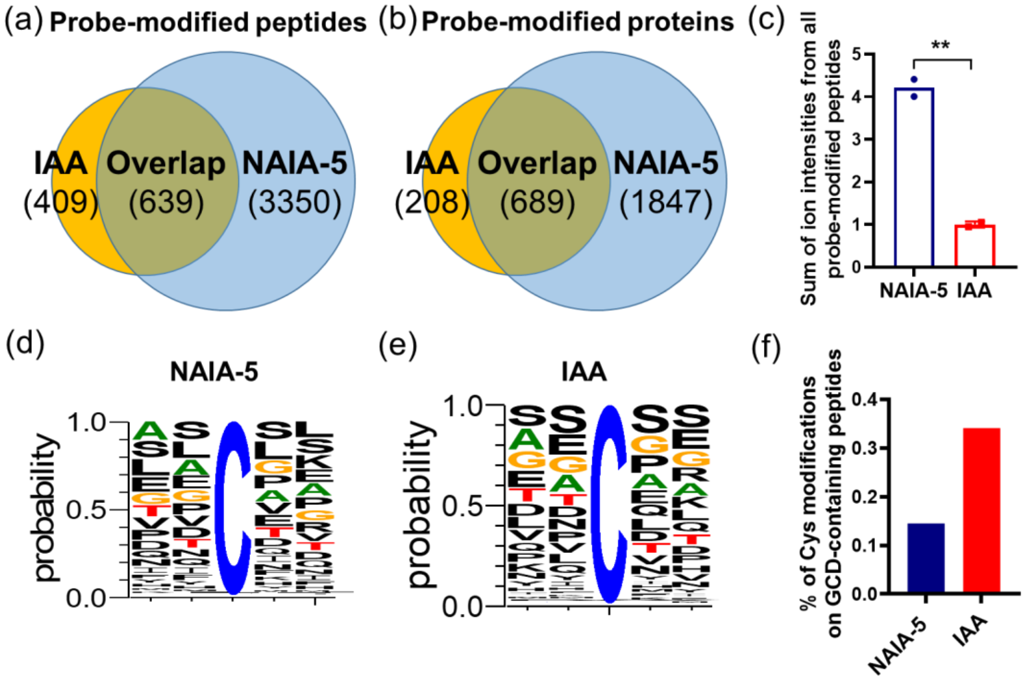

Supplementary Figure 19. MS-based chemoproteomics experiment to investigate Cys profiling in live HepG2 cells by NAIA-5 and IAA. (a and b) Comparison of probe-modified peptides and proteins in live HepG2 cells labeled by NAIA-5 and IAA. (c) Relative intensity of the sum of signals from probe-modified peptides by NAIA-5 or IAA. Quantified data were the average values from $n = 2$ replicates/group. ** $p < 0.01$. (d and e) Local sequence motif of modified Cys by NAIA-5 and IAA respectively. (f) Percentage of Cys modifications on GCD-containing peptides by NAIA-5 and IAA.

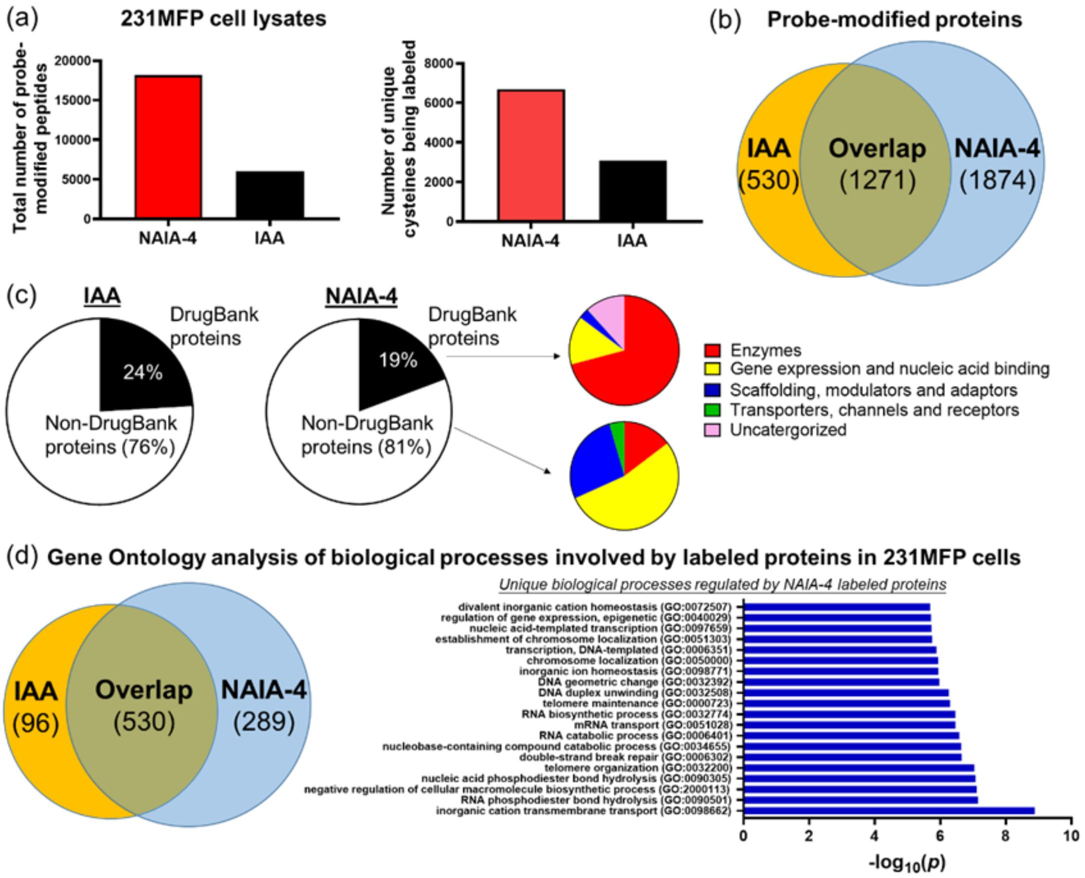

Supplementary Figure 20. LC-MS/MS-based chemoproteomics experiment with Multidimensional Protein Identification Technology (MudPIT) to investigate Cys profiling of 231MFP cell lysates by NAIA-4 and IAA at 100 μM which is the typical working dose of IAA. (a) Total number of probe-modified peptides and number of unique cysteines being identified in the MudPIT experiment. (b) Number of probe-modified proteins identified in the MudPIT experiment. (c) Analysis on probe-modified proteins in 231MFP cells by NAIA-4 and IAA, respectively, using DrugBank database, and analysis on the functions of these proteins by Gene Ontology database. (d) Gene Ontology analysis of the biological processes involved by the probe-modified proteins, with the top 20 unique processes regulated by NAIA-4 shown in the bar chart. P values were determined by Fisher's exact test.

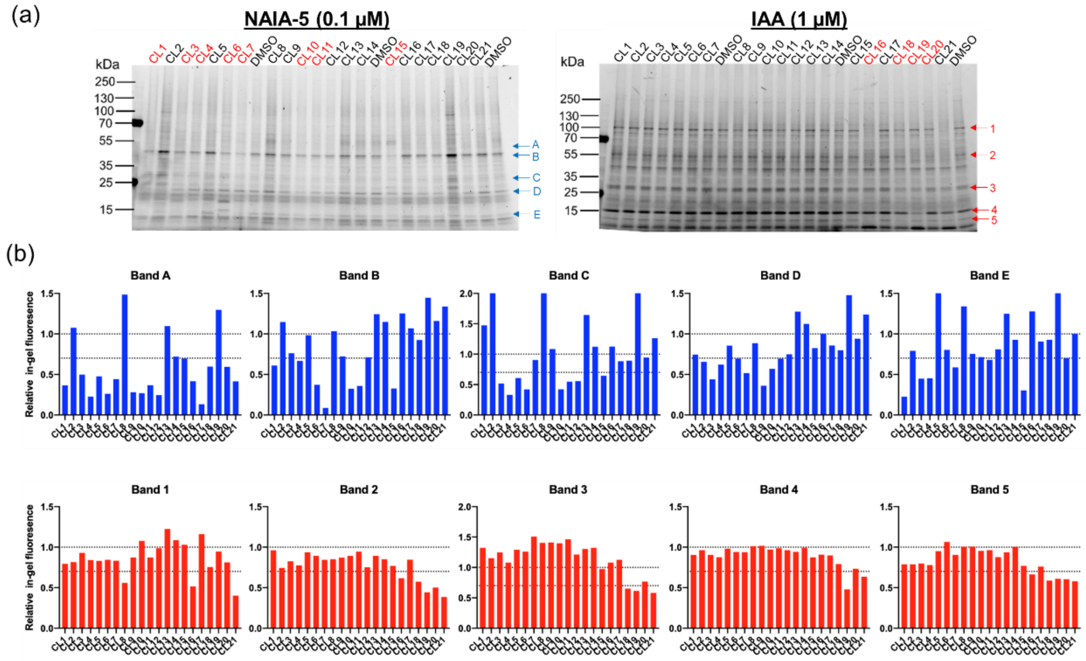

Supplementary Figure 21. Identification of covalent ligands targeting new ligandable cysteines and proteins identified by NAIA-5. (a) Competitive gel-based ABPP experiments for screening out cysteine-reactive covalent ligands (chemical structures can be found in Supplementary Figure 22) that can bind onto functional cysteines in HepG2 cell lysates. Protein bands with lower in-gel fluorescence intensity or band shift indicates binding of the compound onto functional cysteines of the proteins. Note that the labeling efficiency of cell lysate by IAA is lower than that of NAIA-5, so the working concentration of IAA needs to be 10-fold higher for better signal-to-noise. (b) Quantification of fluorescence intensities from the selected protein bands in (a). Hit compounds targeting ligandable cysteines are labeled in red in (a), which are determined from competitive binding (lower than 70% of the fluorescence intensity of DMSO control) in 3 or more bands out of the 5 selected bands. Note that the result was from a single experiment because this experiment was for screening purposes. The hit compound CL1 has been further validated by the dose-dependent experiment, as shown in Supplementary Figure 23, which has 2 replicates/group.

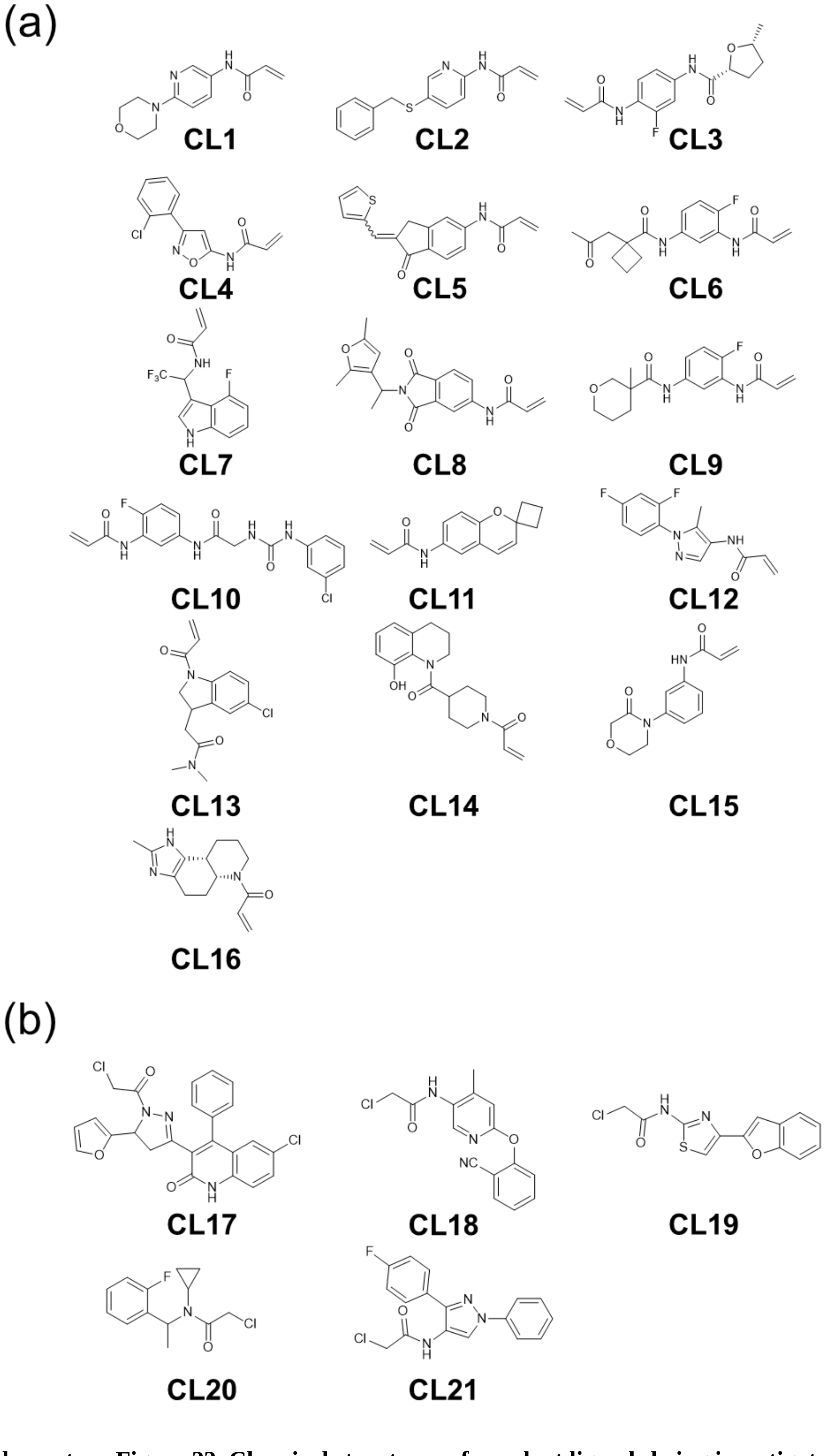

Supplementary Figure 22. Chemical structures of covalent ligands being investigated for their ability to target functional cysteines through competitive ABPP experiments. (a) CL1-16 contain acrylamide warhead, while (b) CL17-21 contain chloroacetamide warhead.

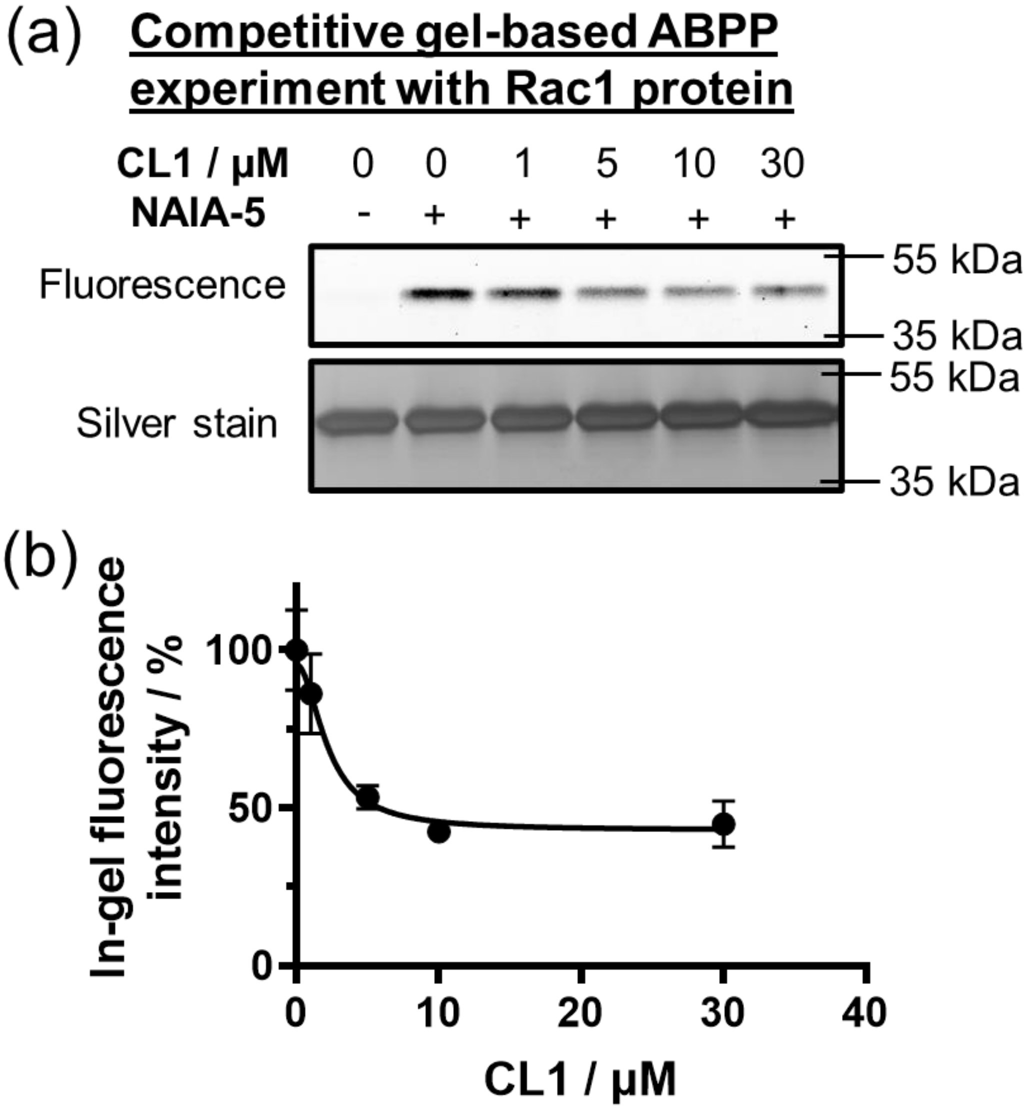

Supplementary Figure 23. Competitive gel-based ABPP experiment to confirm *in vitro* binding of CL1 with Rac1 protein. (a) Purified human GST-Rac1 protein (30 µg) in PBS was incubated with CL1 for 1 h at 37 °C. Then, the solution mixtures were incubated with NAIA-5 (0.1 µM) for 1 h, following by CuAAC reaction with azide-fluor 545 (5 µM). The labeled proteins were then boiled with sampling buffer, and read out by in-gel fluorescence after SDS-PAGE. (b) In-gel fluorescence intensity as determined by ImageJ. Quantified data were the average values from n = 2 replicates/group. It is noteworthy that the in-gel fluorescence intensity did not fall to zero even at 30 µM of CL1 because there are two cysteines on Rac1 capable of labeling by NAIA-5 (Cys105 and Cys178; Supplementary Data 2), while CL1 only bound onto Cys178.

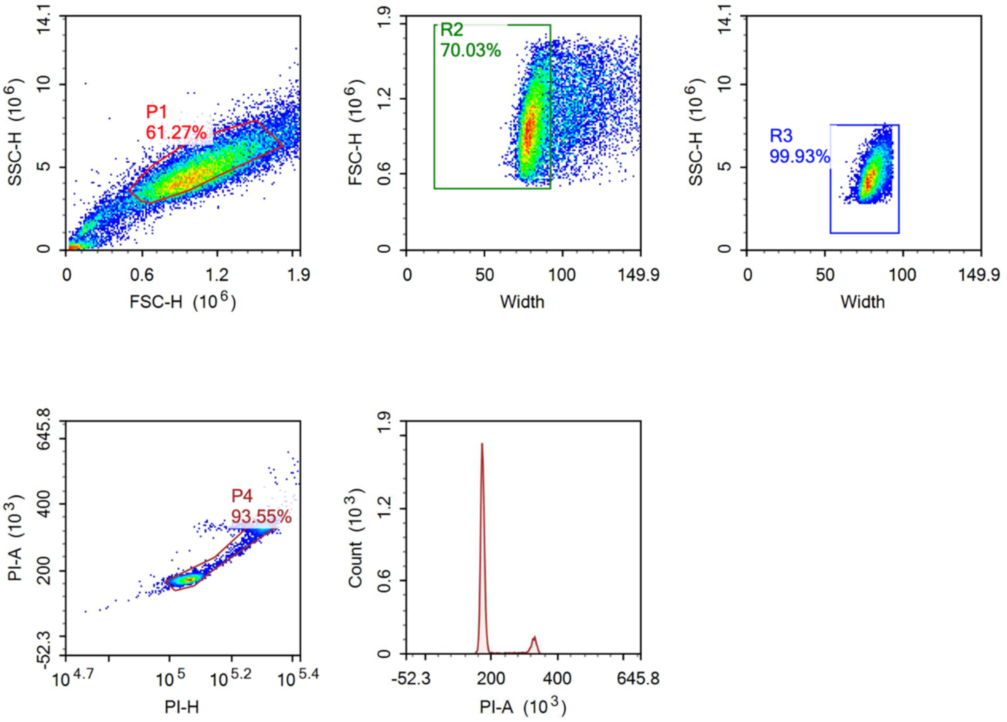

Gate	Count	% Parent	X	Y	Median X	Median Y
All	24,932					
P1	15,277	61.27%	FSC-H	SSC-H	1,028,781	4,704,309
R2	10,698	70.03%	Width	FSC-H	80	976,927
R3	10,690	99.93%	Width	SSC-H	80	4,470,621
P4	10,000	93.55%	PI-H	PI-A	115,321	175,969

Supplementary Figure 24. Representative FACS plots illustrating the gating strategy in studying cell cycle of HepG2 cells. Gate 1 for PI (FSC vs SSC), R2 (Width vs FSC-H), R3 (Width vs SSC-H) and P4 (PI-H vs PI-A) were used to refine cell population. Cell cycle distribution was estimated by Watson Pragmatic algorithm.

^{1}H NMR (400 MHz)

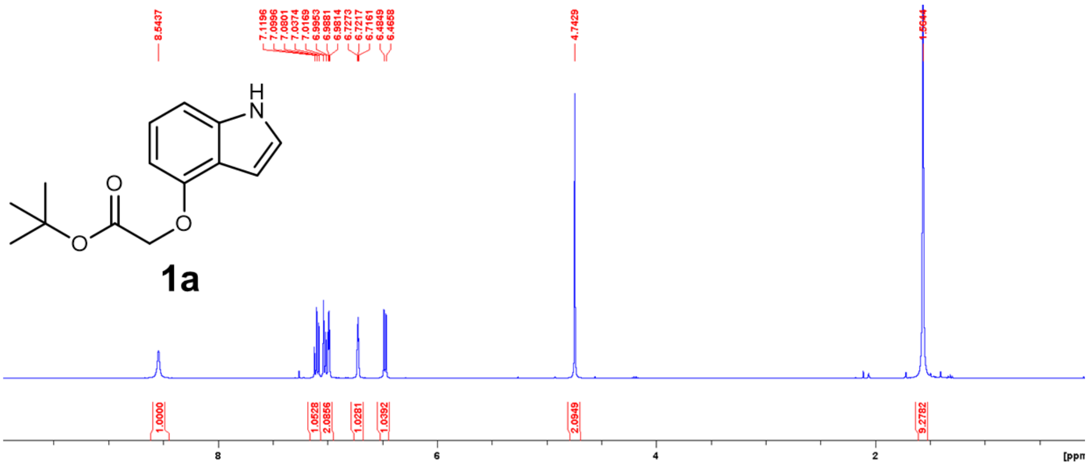

^{13}C{^{1}H} NMR (100 MHz)

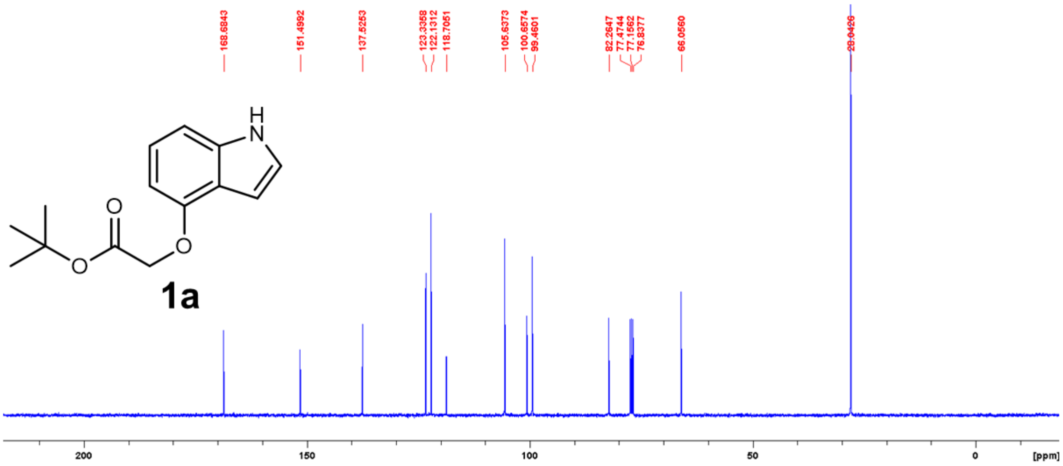

Supplementary Figure 25. ^{1}H and ^{13}C{^{1}H} NMR spectra of compound **1a**.

^{1}H NMR (400 MHz)

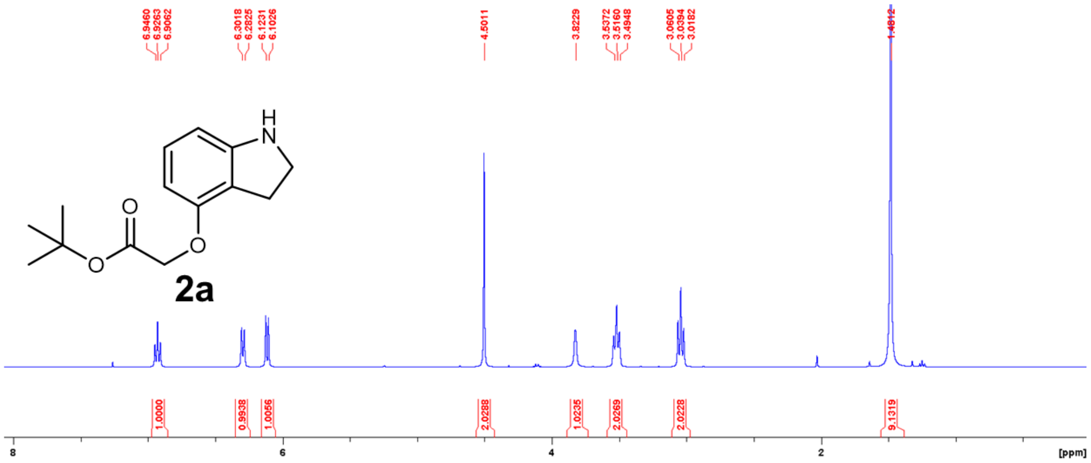

$^{13}C\{^{1}H\}$ NMR (100 MHz)

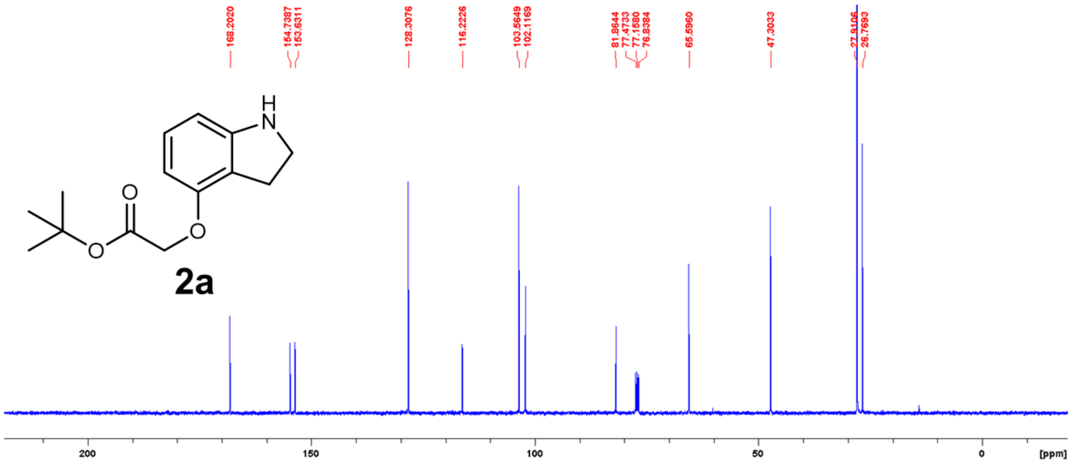

Supplementary Figure 26. ^{1}H and $^{13}C\{^{1}H\}$ NMR spectra of compound **2a**.

^{1}H NMR (300 MHz)

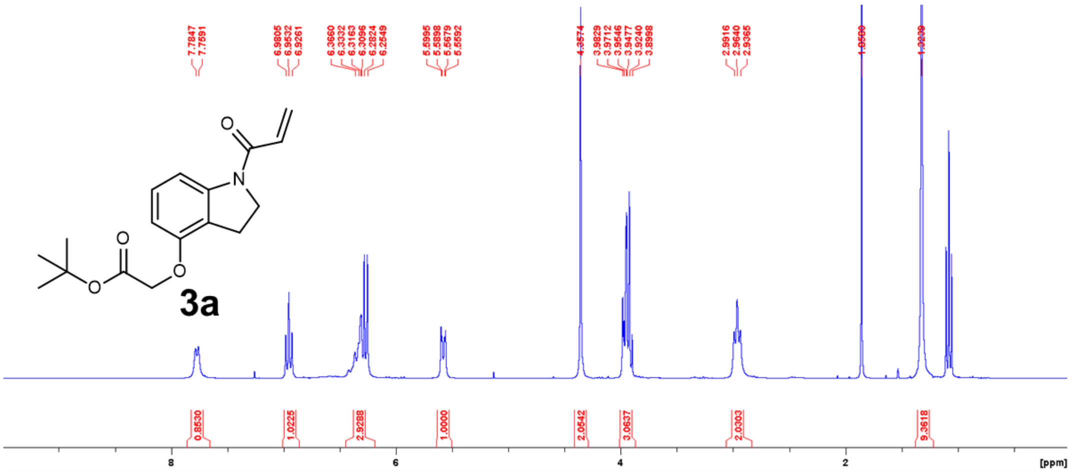

^{13}C{^{1}H} NMR (75 MHz)

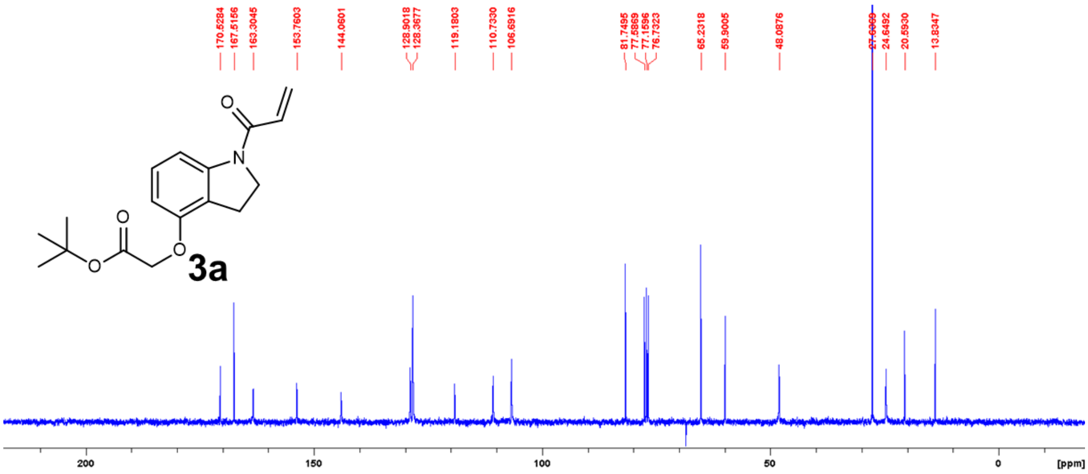

Supplementary Figure 27. ^{1}H and ^{13}C{^{1}H} NMR spectra of compound **3a**.

^{1}H NMR (400 MHz)

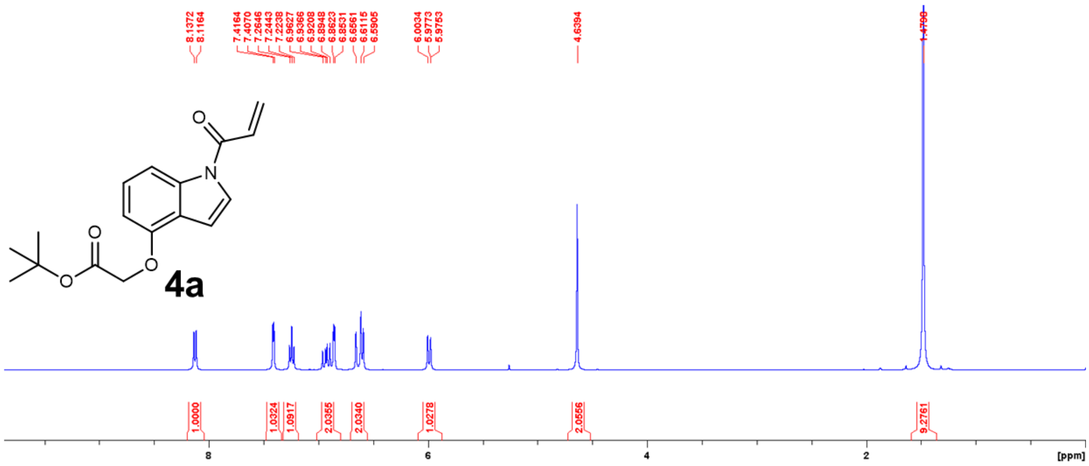

^{13}C{^{1}H} NMR (100 MHz)

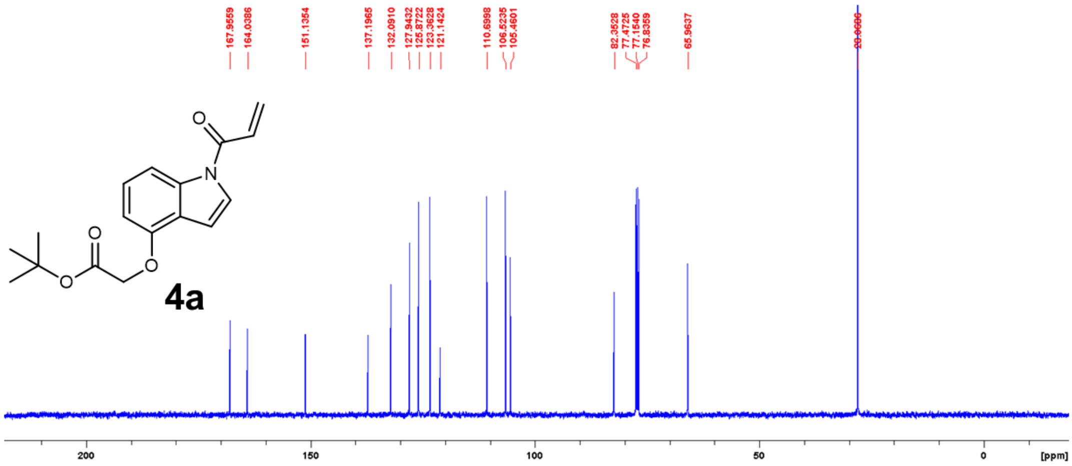

Supplementary Figure 28. ^{1}H and ^{13}C{^{1}H} NMR spectra of compound **4a**.

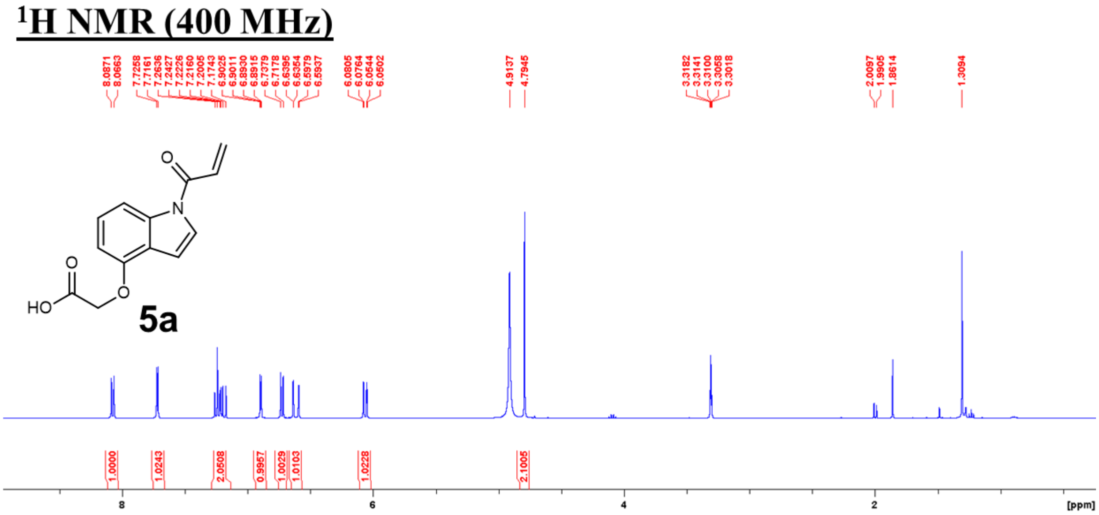

Supplementary Figure 29. ^{1}H NMR spectrum of compound **5a**.

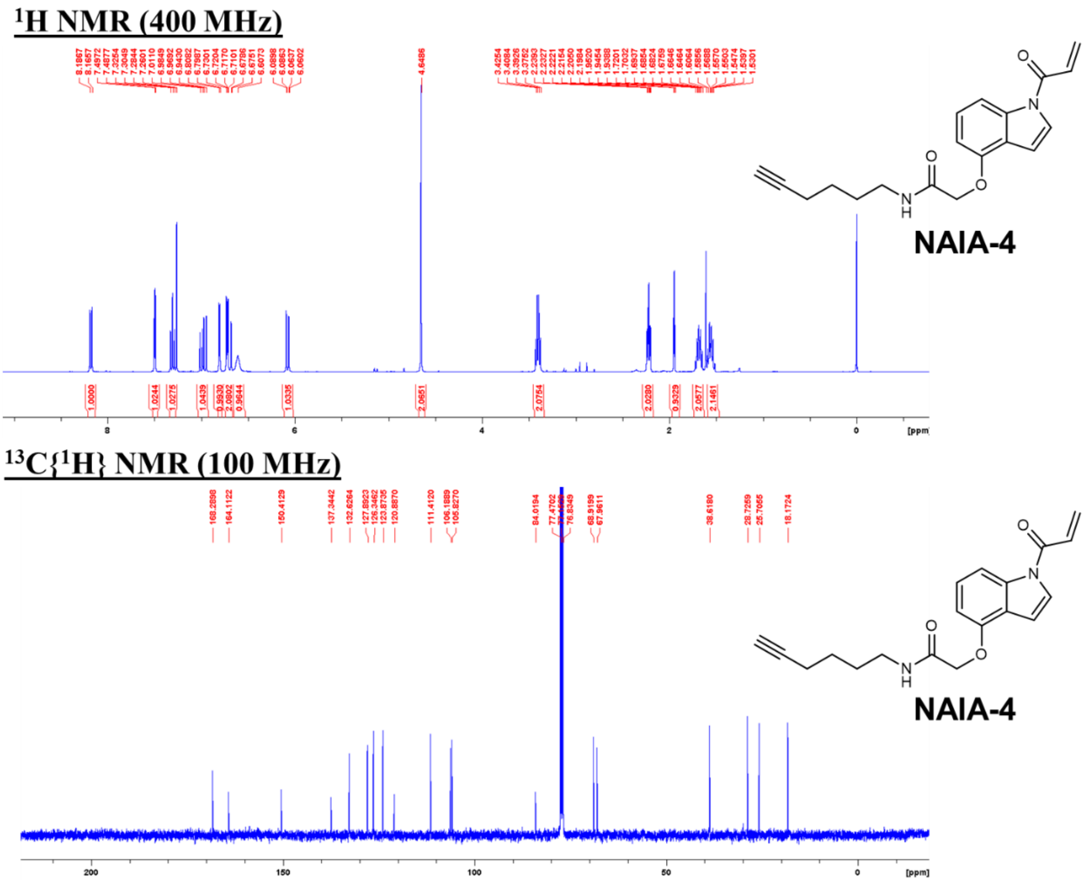

Supplementary Figure 30. ^{1}H and $^{13}C\{^{1}H\}$ NMR spectra of NAIA-4.

^{1}H NMR (500 MHz)

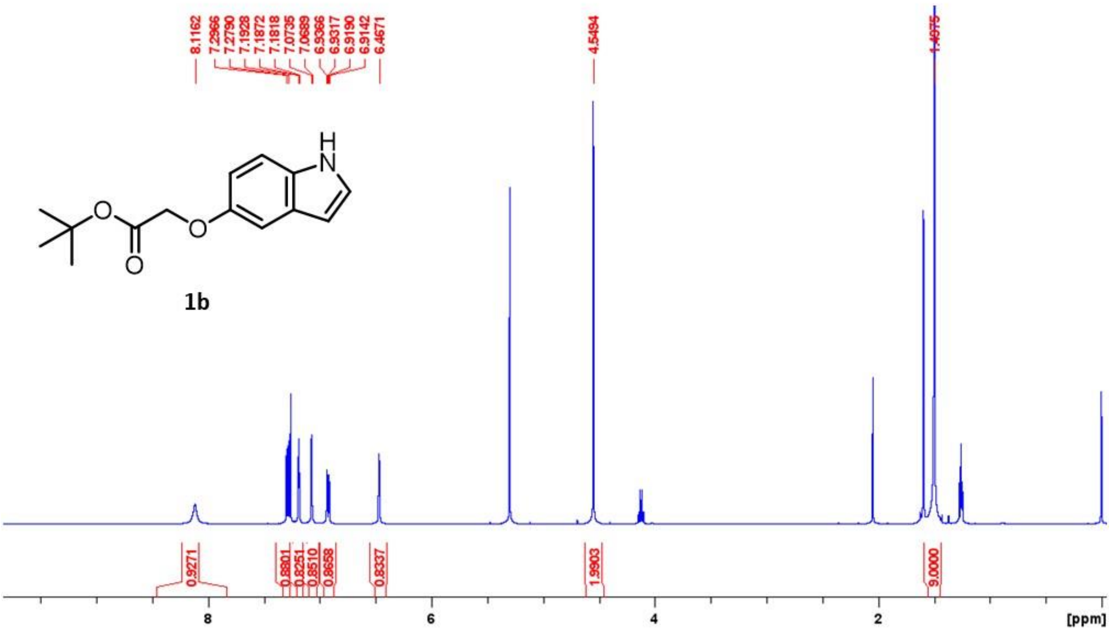

Supplementary Figure 31. ^{1}H NMR spectrum of compound **1b**.

^{1}H NMR (600 MHz)

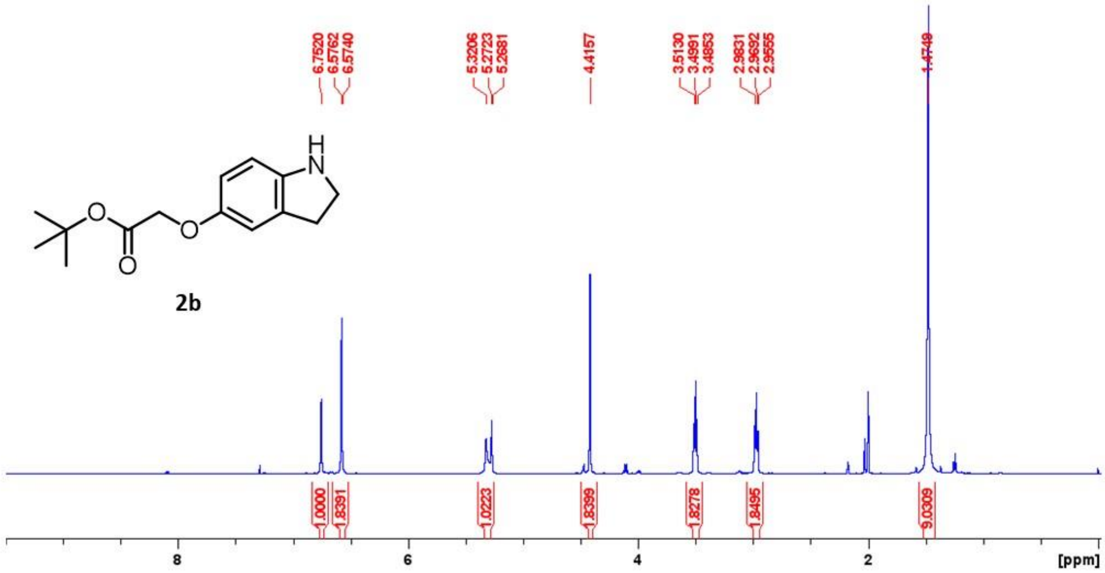

^{13}C NMR (150 MHz)

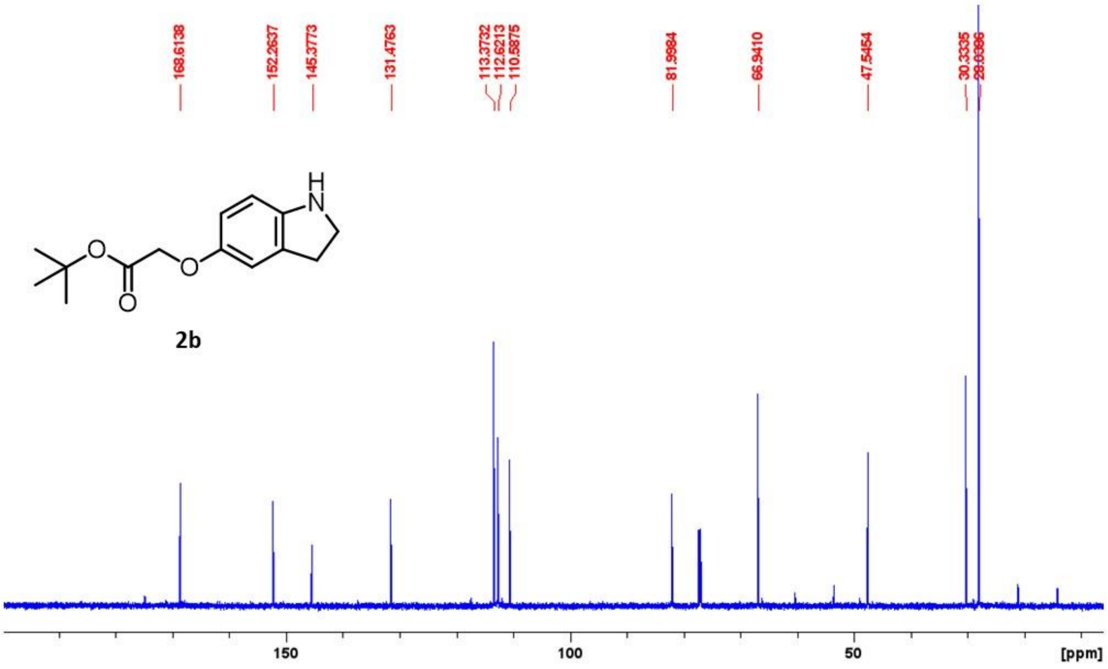

Supplementary Figure 32. ^{1}H and ^{13}C{^{1}H} NMR spectra of compound **2b**.

^{1}H NMR (500 MHz)

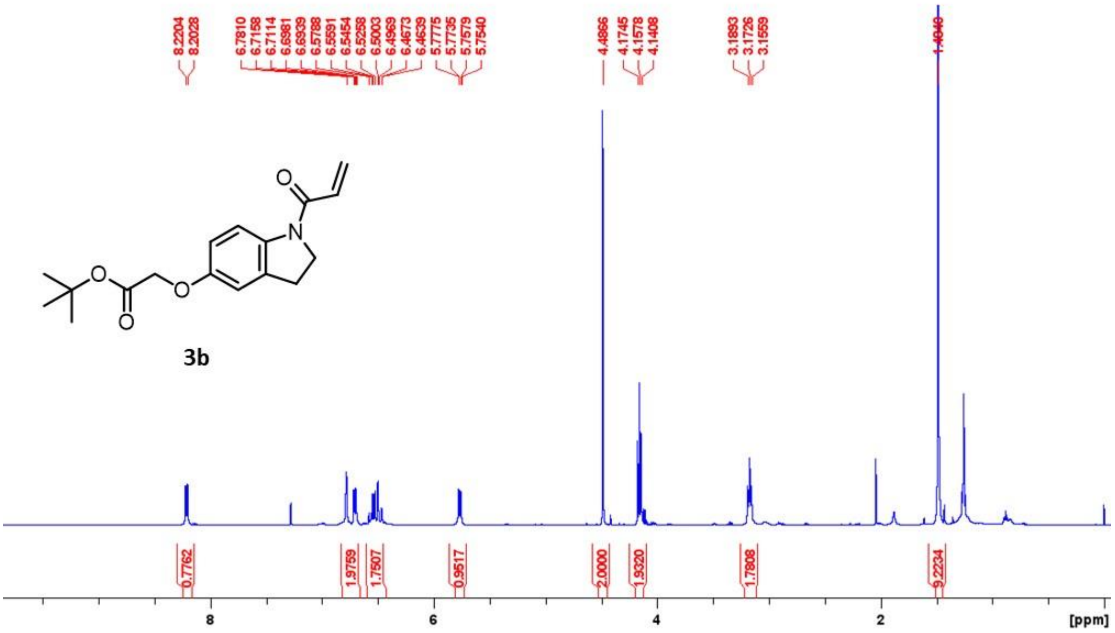

Supplementary Figure 33. ^{1}H NMR spectrum of compound **3b**.

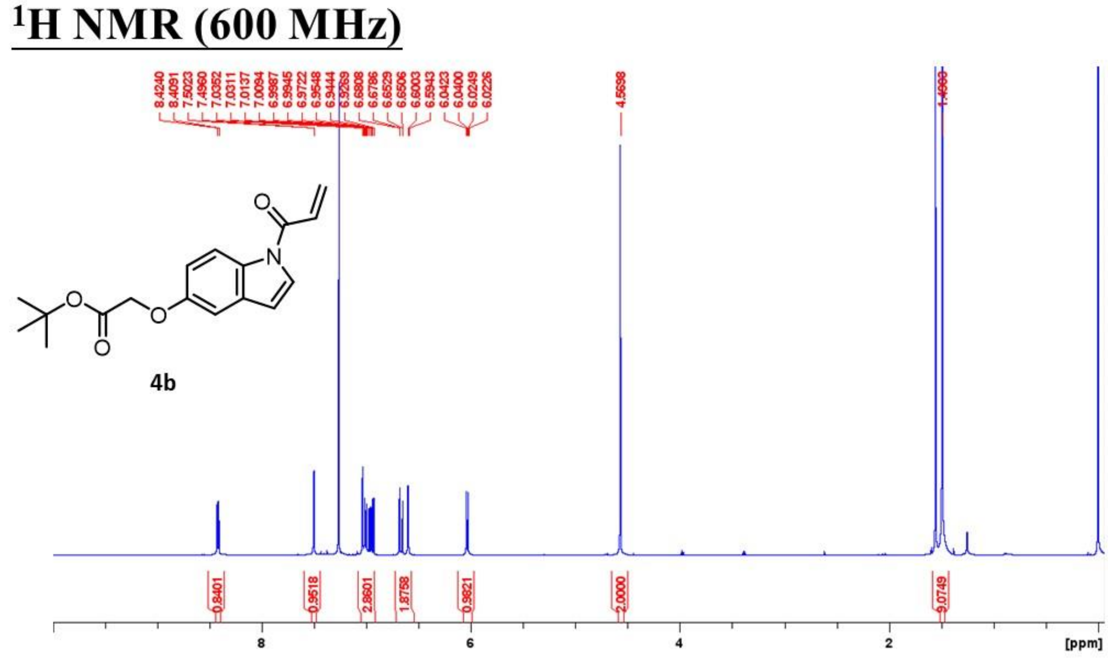

Supplementary Figure 34. ^{1}H NMR spectrum of compound **4b**.

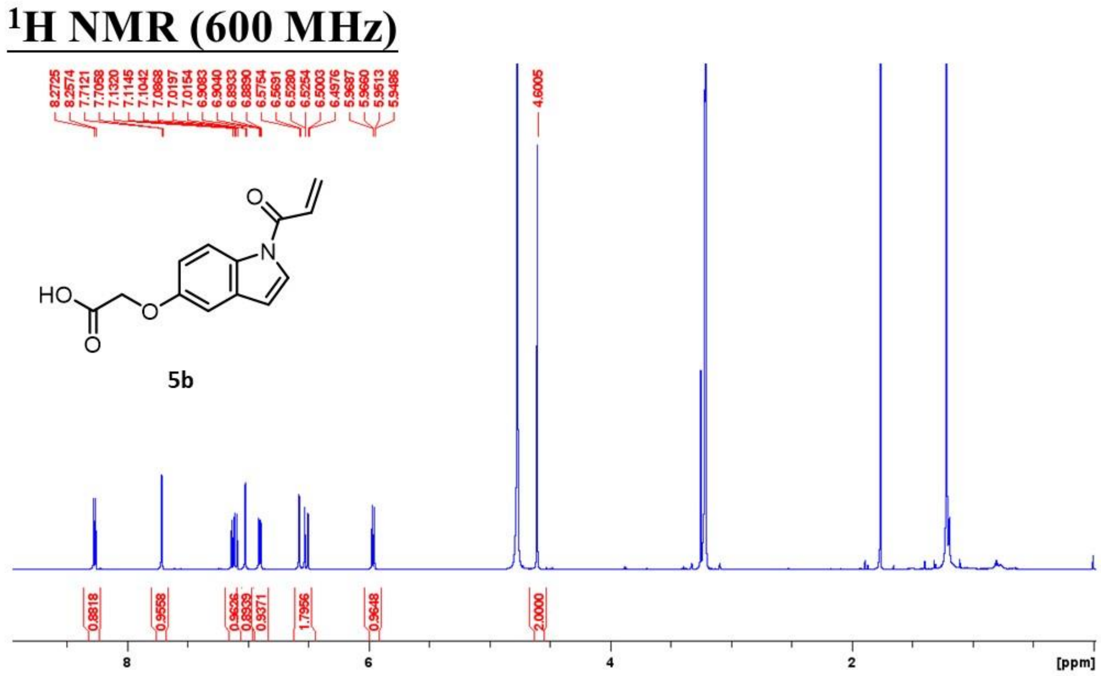

Supplementary Figure 35. ^{1}H NMR spectrum of compound **5b**.

^{1}H NMR (600 MHz)

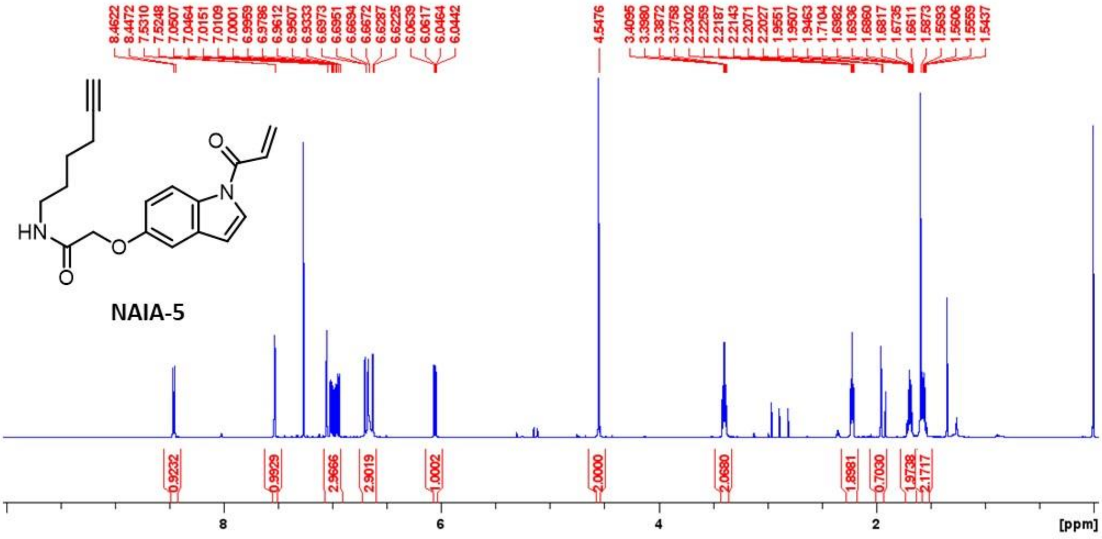

^{13}C NMR (150 MHz)

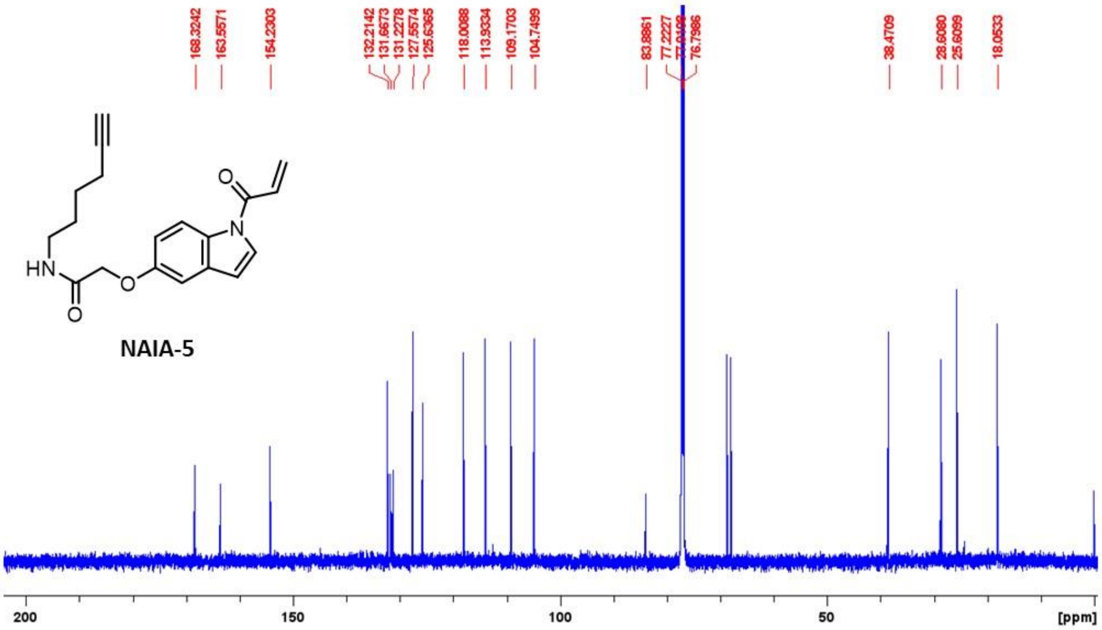

Supplementary Figure 36. ^{1}H and $^{13}C\{^{1}H\}$ NMR spectra of NAIA-5.

Supplementary References

S1. Xu, T. *et al.* ProLuCID: An improved SEQUEST-like algorithm with enhanced sensitivity and specificity. *J. Proteomics* **129**, 16–24 (2015).
