# Supplementary material for: N-Acryloylindole-alkyne (NAIA) enables imaging and profiling new ligandable cysteines and oxidized thiols by chemoproteomics
Source: Nat Commun. 2023 Jun 15;14:3564. doi: 10.1038/s41467-023-39268-w (PMC10272157; doi:10.1038/s41467-023-39268-w)
Supplement: Supplementary file 3 — Description of Additional Supplementary Files [file 41467_2023_39268_MOESM3_ESM.pdf]

Description of Additional Supplementary Files

File Name: Supplementary Data 1
Description: NAI/NAIA-5 couple to profile oxidized cysteines in HepG2 cells

File Name: Supplementary Data 2
Description: NAIA-5 vs IAA to profile functional cysteines in HepG2 cell lysates

File Name: Supplementary Data 3
Description: NAIA-5 vs DBIA to profile functional cysteines

File Name: Supplementary Data 4
Description: Comparison of functional cysteines profiled by NAIA-5 with those reported in CysDB

File Name: Supplementary Data 5
Description: NAIA-5 vs IAA to profile functional cysteines in live HepG2 cells

File Name: Supplementary Data 6
Description: NAIA-4 vs IAA to profile functional cysteines in 231MFP

File Name: Supplementary Data 7
Description: Competitive ABPP experiment using NAIA-5 and CL-Sc to identify ligandable cysteines

File Name: Supplementary Data 8
Description: Competitive ABPP experiment using NAIA-5 to identify protein targets of CL1 in HepG2
